# Atypical Development of Attentional Control Associates with Later Adaptive Functioning, Autism and ADHD Traits

**DOI:** 10.1007/s10803-020-04465-9

**Published:** 2020-03-27

**Authors:** Alexandra Hendry, Emily J. H. Jones, Rachael Bedford, Linn Andersson Konke, Jannath Begum Ali, Sven Bӧlte, Karin C. Brocki, Ellen Demurie, Mark Johnson, Mirjam K. J. Pijl, Herbert Roeyers, Tony Charman, the Eurosibs Team, Sheila Achermann, Mary Agyapong, Rebecka Astenvald, Lisa Axelson, Tessel Bazelmans, Karlijn Blommers, Chloè Bontinck, Carlijn van den Boomen, Sofie Boterberg, Ricarda Braukmann, Yvette de Bruijn, Eva Bruyneel, Jan K. Buitelaar, Leila Dafner, Fahime Darki, Kim Davies, Mutluhan Ersoy, Terje Falck-Ytter, Janice Fernandes, Zoë Freeman, Teea Gliga, Gustaf Gredebäck, Marian Greensmith, Rianne Haartsen, Sanne van Ierland-Veerhoek, Maretha V. de Jonge, Sarah Kalwarowsky, Chantal Kemner, Anna Kolesnik, Manon de Korte, Johan Lundin-Kleberg, Nicolette M. Munsters, Pär Nyström, Greg Pasco, Laura Pirazzoli, Johanna Ristolainen, Andrietta Stadin, Chloë Taylor, Emilia Thorup, Natalie vaz, Loes Vinkenvleugel, Emma Ward, Petra Warreyn, Lilli N. van Wielink

**Affiliations:** 1 Department of Experimental Psychology, University of Oxford, Oxford, UK; 2 Psychology Department, Institute of Psychiatry, Psychology & Neuroscience, King’s College London, London, UK; 3 Centre for Brain and Cognitive Development, Birkbeck College, University of London, London, UK; 4 Biostatistics Department, Institute of Psychiatry, Psychology & Neuroscience, King’s College London, London, UK; 5 Department of Psychology, Uppsala University, Uppsala, Sweden; 6 Center of Neurodevelopmental Disorders (KIND), Centre for Psychiatry Research, Department of Women’s and Children’s Health, Karolinska Institutet & Child and Adolescent Psychiatry, Stockholm Health Care Services, Region Stockholm, Stockholm, Sweden; 7 Curtin Autism Research Group, School of Occupational Therapy, Social Work and Speech Pathology, Curtin University, Perth, WA Australia; 8 Department of Experimental Clinical and Health Psychology, Ghent University, Ghent, Belgium; 9 Department of Psychology, University of Cambridge, Cambridge, UK; 10 Radboud University Nijmegen Medical Centre, Donders Institute for Brain, Cognition and Behaviour, Karakter Child and Adolescent Psychiatry University Centre, Nijmegen, The Netherlands

**Keywords:** Autism, ADHD, Attention, Atypical development, Infant, Intermediate phenotype

## Abstract

Autism is frequently associated with difficulties with top-down attentional control, which impact on individuals’ mental health and quality of life. The developmental processes involved in these attentional difficulties are not well understood. Using a data-driven approach, 2 samples (*N* = 294 and 412) of infants at elevated and typical likelihood of autism were grouped according to profiles of parent report of attention at 10, 15 and 25 months. In contrast to the normative profile of increases in attentional control scores between infancy and toddlerhood, a minority (7–9%) showed plateauing attentional control scores between 10 and 25 months. Consistent with pre-registered hypotheses, plateaued growth of attentional control was associated with elevated autism and ADHD traits, and lower adaptive functioning at age 3 years.

**Electronic supplementary material:**

The online version of this article (10.1007/s10803-020-04465-9) contains supplementary material, which is available to authorized users.

Although the core diagnostic criteria for Autism Spectrum Disorder (ASD) focus on social-communication atypicalities and rigid and repetitive behaviours that cause impairment (APA 2013), the autistic spectrum encompasses a broad range of behavioural characteristics. For example, autistic individuals vary considerably in the extent of autism-related traits, in their intellectual ability, and in their ability to function independently in day-to-day life (Szatmari et al. 2015; Visser et al. 2017). Despite the heritability of autism (Tick et al. 2016), attempts to find a single genetic account of autism have largely failed; likely due in part to heterogeneity within the spectrum (Feczko et al. 2019; Happé et al. 2006), and in part to overlap with other conditions. Around 71% of children with an autism diagnosis also meet criteria for at least one other condition; most commonly social anxiety disorder, Oppositional Defiant Disorder and Attention Deficit Hyperactivity Disorder (ADHD) (Simonoff et al. 2008).

A more fruitful approach to developing our understanding of the etiology of autism, and why it so frequently co-occurs with other conditions, may be to focus on developmental pathways within functional domains. The Research Domain Criteria (RDoC) is a research classification system based on dimensional characteristics of behaviour which are grounded in neurobiology (Cuthbert and Insel 2013). This approach looks beyond diagnostic ‘symptoms’ to transdiagnostic systems or intermediate phenotypes; quantifiable processes that are interposed between gene and clinical phenotype and which might contribute to multiple neurodevelopmental conditions (Meyer-Lindenberg and Weinberger 2006). One domain of interest within RDoC is attention, which can be characterised in terms of controlled (i.e. ‘top-down’) versus automatic (i.e. ‘bottom-up’) attention (NIMH 2016). In this proof-of-principle application of the RDoc approach we focus particularly on top-down attentional control, which encompasses both the ability to sustain attention (and inhibit distractibility) and endogenous shifting of attention. Other RDoC domains which may be of relevance to autism and ADHD, such as social communication, state regulation (i.e. autonomic arousal) and perception, are beyond the scope of this study—but it is worth noting that top-down attentional control likely interacts with bottom-up perceptual and arousal processes in the context of autism and attention development (Bast et al. 2018; Hendry et al. 2019; Karalunas et al. 2014).

Autism has been linked to atypical attentional control at the behavioural and neural level (Fan 2013; Murray et al. 2005). Furthermore, difficulties with sustaining attention are of obvious relevance to Attention Deficit Hyperactivity Disorder (ADHD), particularly the inattentive and combined subtypes (APA 2013). As previously noted, ADHD frequently co-occurs with autism (Sokolova et al. 2017). Autism and ADHD may share common antecedents of genetic origin, relating to difficulties with attentional control (Visser et al. 2016). This common genetic link may explain why autism and ADHD cluster in families, such that a child with an autistic first-degree relative is more likely than average to have ADHD, and vice versa (Chien et al. 2017; Ghirardi et al. 2017; Miller et al. 2019; Oerlemans et al. 2015; Septier et al. 2019). Thus, in this study we investigate early development of attentional control as a possible intermediate phenotype of autism and ADHD.

Whilst understanding the role of attentional control in the etiology of autism and ADHD is of research interest in itself, understanding how variation in the development of attentional control supports and restricts education, life and social-skills outcomes is likely to be of even greater interest and real-world benefit to autistic people and those who love them (Cusack and Sterry 2016). There is reason to believe that attentional control may be particularly relevant to quality of life and to adaptive functioning (i.e. the ability to perform everyday functions such as listening to instructions and playing co-operatively with others, and to cope with a range of situations and tasks, such as self care, at an age-appropriate level of independence): Autistic children with co-occurring ADHD experience greater impairment in adaptive functioning and health-related quality of life compared with autistic children who present without clinical levels of ADHD symptoms (Sikora et al. 2012; Yerys et al. 2009). If, as we reason above, attentional control is contributing to the overlap between autism and ADHD, it is feasible that the specific adaptive functioning difficulties observed for children with both autism and ADHD may be attributable to difficulties with attentional control. However, research on this topic to date has been primarily cross-sectional, involving older children and adults, and has rarely considered attentional control as an intermediate phenotype of autism and ADHD (Jonsson et al. 2017); a gap we seek to address in this study.

## Investigating Early Development of Attentional Control

The behavioural phenotypes of autism and ADHD are each influenced by numerous interactions and feedback loops between multiple genetic and environmental influences across development (Visser et al. 2016). It is therefore possible that the attention difficulties frequently observed in autistic adults and their relatives are a consequence of atypical interaction with the world during earlier development. To better understand whether attentional control is an intermediate phenotype of autism and ADHD irrespective of these feedback loops we need to monitor relevant behaviours from as early as possible postnatally, before they are overly influenced by intellectual ability, compensatory or secondary mechanisms, and interactions with co-occurring conditions (Johnson et al. 2015). The ability to consciously control one’s own attention emerges prior to a baby’s first birthday, continues to develop rapidly in infancy and toddlerhood, and acts as a foundation for the development of more-complex cognitive processes (Hendry et al. 2016, 2019). Thus, the first 3 years of life is an important focal point for research into the development of attentional control.

One way to investigate the development of attentional control in infancy and toddlerhood is through parent report. Parent report provides a cost- and time-effective insight into infant behaviour that is both broad and deep (Hedge et al. 2017; Putnam et al. 2006; Rothbart and Mauro 1990). Parents are ideally positioned to report on low-frequency behaviours that may be difficult to capture in a laboratory. Further, since generalisation of skills from one context to another can cause particular challenges for some children (Wong et al. 2007), parent report can be valuable in ascertaining whether or not behaviours are pervasive across contexts.

Parent report of attention across the first years of life is characterised by both change and stability. From around 9 months of age, the pattern in typical development is for parent-reported attentional control scores to increase with age (Gaertner et al. 2008; Putnam et al. 2006). Yet stability in individual differences is nested within this developmental progression. Whilst Putnam et al. (2008) report only weak correlations between infant and toddler attentional control scores, this may be attributed to the wide age-span for their infant group which crosses the 7- to- 9-month mark, considered to be a critical period of transition in attention development (Hendry et al. 2019). By toddlerhood, longitudinal stability correlations for attentional control scales are moderate-to-large across spans of 6–18 months (Gaertner et al. 2008; Putnam et al. 2006).

Furthermore, there is evidence for a predictive association between parent report of attentional control in infancy and childhood behavioural difficulties, even in general community samples. Low parent-reported attentional focus scores at age 18–32 months are associated with elevated internalising and externalising problems at 37–59 months (Gartstein et al. 2012), whilst individual differences at 10 months of age in a composite attention-regulatory measure are predictive of ADHD-related behaviours at age 3 years (Frick et al. 2019). In combination, these studies indicate that infant attentional control is measurable via parent report, stable, and shows associations with later phenotypic variation.

## Current Evidence for Atypical Development of Attentional Control in Autism and ADHD

Community diagnoses of ASD for children under age 3 years are rare, so infant-sibling designs have been used to study infants who may be on a pathway to autism. Infant-sibling designs work on the premise that infants with an older brother or sister with an ASD diagnosis are more likely [henceforth at Elevated Likelihood (EL)] to develop clinically-significant autism traits themselves, compared with infants with an older sibling and no family history of autism [henceforth at Typical Likelihood (TL)]. Current estimates suggest that EL infants have around a 20% likelihood of receiving a diagnosis of ASD (Messinger et al. 2015)—as opposed to the community prevalence rate of around 1.7% (Baio et al. 2018). Further, EL infants who do not show clinical levels of ASD symptoms by age 3 years have around a 30% likelihood of showing elevated autism traits, and/or lower adaptive functioning than age-matched TL peers (Charman et al. 2017).

In contrast to the clear evidence for parent-reported difficulties with attentional control amongst children aged 2 years or older already with an ASD diagnosis (Adamek et al. 2011; Konstantareas and Stewart 2006; Macari et al. 2017), evidence from infant-sibling studies for autism-related differences in attentional control in children aged 2 years and below is mixed. In one preliminary study, EL infants showing elevated autism traits at 24 months were found to have *higher* parent-reported scores on the Duration of Orienting scale at 12 months compared with EL infants who did not reach diagnostic criteria, and with TL children (Zwaigenbaum et al. 2005). However, the sample size was very small, with only 6 infants in the elevated-traits group, and this finding has not been replicated. Subsequently, researchers have tended to find lower parent-reported attentional control scores amongst EL 2-year-olds in general (Garon et al. 2016), and those EL infants later diagnosed with ASD specifically (Garon et al. 2009). However, Clifford et al. (2013) found no evidence for group differences in attentional control at 14 or 24 months, either in terms of diagnostic outcome or familial history of ASD. Further, whilst 16- to 36-month-olds with an early diagnosis of ASD were reported by parents to have greater difficulty in attentional shifting compared to both typically-developing and developmentally-delayed comparison groups, and a diminished ability to focus attention compared with the typically-developing group only—neither variable showed significant associations with autism traits (Macari et al. 2017). One reason for this mixed literature may be that early difficulties with attentional control amongst children on a pathway to autism are masked by the heterogeneity characteristic of EL populations.

To our knowledge, only one study has investigated the associations between standardised parent report of attentional control in infancy and later ADHD traits amongst infants with a familial history of autism, and that study found no evidence for a predictive association from attentional focus scores at 7, 14 or 24 months and ADHD traits in mid childhood (Shephard et al. 2019). However, others have found that EL infants with clinical traits of ADHD but not ASD at age 8–11 years showed higher levels of broad parent-reported behavioural or temperament concerns (spanning high activity level, poor attention, behavioural dysregulation and difficulties in mood/general disposition) at ages 2 and 3 years (but not at 6- to 18-months) compared with TL and EL peers (Miller et al. 2016). Given the evidence referred to above, indicating that ADHD and autism do share common antecedents of genetic origin relating to difficulties with attentional control, we argue that further research is merited: firstly to understand whether early disruption to development of attentional control can be detected using methodological approaches suited to capturing heterogeneous developmental processes amongst infants with a familial history of autism; and secondly to better understand the implications of such disruption.

## Capturing Change and Heterogeneity

In order to detect early evidence for disruption to attentional processes, taking a developmental approach may be key. Colombo and colleagues have argued that changes in looking behaviour towards the end of the first year of life and into the second year are sensitive to the development of attentional control (Colombo and Cheatham 2006). Consistent with this, we have shown in a sample of EL and TL infants that changes in looking behaviour between 9 and 15 months are predictive of parent-reported effortful control (a composite of attentional and impulse control, low intensity pleasure and cuddliness scores) at age 3 years (Hendry et al. 2018). Moreover, we found that EL infants as a group showed an attenuated change in peak look durations to faces. Meanwhile Miller et al. (2018) found that EL infants with clinical levels of ADHD traits in mid childhood showed a plateau in overall looking time to screen during 2 eye-tracking tasks, in contrast to the TL profile of increases in sustained attention between 3 and 24 months of age.

Accounts of early developmental change may be improved still further by considering sub-group differences in development. For example, Bussu et al. (2019) and Sacrey et al. (2019) have identified separable trajectories of adaptive functioning between the ages of 8 and 36 months amongst EL and TL infants. In neither case was a diagnostic outcome of autism associated uniquely with one trajectory; i.e. autistic children showed heterogeneity of development. To our knowledge, this approach has not yet been applied to parent report of attention—yet the identification of intermediate phenotypes may be best served through using data-driven approaches in this way.

## The Current Study

In the current study, we investigate attentional control as a possible intermediate phenotype of autism and ADHD, using an analytic approach sensitive to heterogeneity in early development. Specifically, we use Latent Class Analysis (LCA) to derive data-driven subgroups relating to parent-reported attentional control between 10 and 25 months. We then test the association between data-driven profiles of attentional control development and adaptive functioning and autism and ADHD traits, at age 3 years.

LCA is a specific form of mixture modelling which aims to recover homogeneous subpopulations from within a heterogeneous sample, based on the means of observed variables (Lanza et al. 2013). The underlying rationale behind LCA is thus similar to k-means clustering, but LCA offers 2 main advantages: firstly LCA estimates the probability of each observation falling into a particular class, and thus allows us to account for some uncertainty; secondly, LCA does not assume equal variances across variables—an assumption that is often violated in developmental research. LCA also offers an advantage over another variant of mixture modelling, Latent Growth Curve Modelling, in that it provides a means of identifying differences in developmental profiles without assuming a consistent underlying construct over time (McCutcheon 1987). This is key when considering parent report of attention across the first 3 years of life as the best-established parent-report questionnaire uses a single scale to capture attentional control in early infancy (Gartstein and Rothbart 2003), but is replaced by a more-nuanced measure in toddlerhood, with specific and dissociable scales for attentional focus and attention shifting (Putnam et al. 2006).

To be able to detect relatively-rare subgroups with LCA, large samples are required—yet research with special populations such as EL infants has tended to rely on relatively small samples to date (Jones et al. 2014). By pooling data from multiple studies, we are able to achieve the sample sizes required, with a further advantage of increasing the generalizability of the sample. We use data from both EL and TL infants as a means of maximising variation in attentional control, but analyses are run without a priori allocation of familial likelihood or diagnostic outcome to avoid reifying diagnostic criteria. Below we present results from a discovery sample of 294 UK-based EL and TL infants, and a pre-registered (https://osf.io/4afq9) sample of 412 EL and TL infants from Sweden, Belgium, the Netherlands, and the UK. By presenting both exploratory and pre-registered confirmatory analyses from 2 separate large samples we aim to contribute to efforts to improve the robustness and replicability of developmental research (Davis-Kean and Ellis 2019).

## Method

### Participants

#### Sample 1: Discovery Sample

Data were collected from 3 longitudinal cohorts between 2006 and 2018 as part of the British Autism Study of Infant Siblings (see Supplementary Materials 1 (SM1) for further details): Parent-report of temperament data from some of these cohorts has previously been reported by Clifford et al. (2013) (Phase 1 only) and Pijl et al. (2019) (Phases 1 and 2 only) but did not include a longitudinal analysis of scales specifically relating to control of attention. Of the 301 infants recruited, 219 (113 males) were categorised as EL for ASD on the basis of having 1 or more older siblings with a community clinical diagnosis of ASD (see SM1 for further details). The remaining 82 infants (36 males) were categorised as TL controls. TL infants were recruited from a volunteer database at the Birkbeck Centre for Brain and Cognitive Development. These infants were full-term (with one exception), had normal birth weight, and had no first-degree family members with ASD (as confirmed through parent interview regarding family medical history). All had at least 1 older-sibling (half-sibling/s in 3 cases).

#### Sample 2: Confirmatory Sample

Data were collected from 4 longitudinal cohorts: the Early Autism Sweden (EASE) project in Sweden (*n* = 175), the Babystudie project in Belgium (*n* = 96), the Sisters and Brothers of Children with Autism (ZEBRA) project in the Netherlands (*n* = 99), and the Studying Autism and ADHD in at Risk Siblings (STAARS) project in the UK (excluding infants whose 10-month visit was prior to December 2018, as they were included in Sample 1, *n* = 72). None of the Sample 2 parent-report of attention data has been previously published. Of the 441 infants recruited, 278 (130 males) were categorised as EL for ASD on the basis of having 1 or more first-degree biological relatives with a community clinical diagnosis of ASD. The remaining 163 infants (83 males) were categorised as TL controls. TL infants were recruited through volunteer databases, social media, and well-baby clinics. Inclusion criteria for TL infants were full-term birth and no ASD within first-degree relatives.

#### Age Inclusion Criteria and Final Sample Size

For both samples, infants were assessed with multiple visits. To ensure that measures captured a comparable point in development at each time-point, inclusion constraints were set to span a 3-month period for the first 2 time-points (in infancy, when attentional control is still undergoing rapid change), and a 6-month period for the third time-point (in toddlerhood, when individual differences in attentional control are more stable) (Putnam et al. 2008). These criteria were pre-registered for Sample 2. The final sample size and age ranges are presented in Table 1. A total of 294 infants (212 EL) contributed data to at least 1 time-point for Sample 1, and 412 infants (261 EL) contributed data to at least 1 time-point for Sample 2. The aim of including a larger sample in Sample 2 compared with Sample 1 was to increase generalisability and to establish the robustness of the class decomposition; we check this still further in SM3 by applying the main analyses to Samples 1 and 2 combined.

**Table 1:** Age in months by likelihood group for each time-point

	10-month time-point M (SD)		15-month time-point M (SD)		25-month time-point M (SD)	
Sample	1	2	1	2	1	2
Elevated likelihood	9.28 (.86) *n* = 155	10.31 (.87) *n* = 207	15.12 (1.00) *n* = 182	14.53 (1.04) *n* = 92	25.61 (1.45) *n* = 156	24.47 (1.08) *n* = 156
Typical likelihood	9.17 (.70) *n* = 41	10.13 (.57) *n* = 139	15.20 (.98) *n* = 61	14.48 (.58) *n* = 68	24.74 (.98) *n* = 74	24.93 (1.36) *n* = 101

### Measures

#### Control of Attention

As part of the protocol for the larger studies incorporating experimental tasks (not reported here), parents were asked to report on a range of aspects of their child’s temperament, including the child’s attentional behaviour during the previous week (10 and 15 months) or fortnight (25 months). At the 10- and 15-month time-points, the Duration of Orienting scale of the Infant Behavior Questionnaire (IBQ) (Rothbart 1981) or IBQ-Revised (IBQ-R) (Gartstein and Rothbart 2003) was used as our measure of attentional control (see SM1 for discussion of 1 variation from this pre-registered plan in the Swedish cohort in Sample 2, and SM3a for the results of analysis with the Swedish cohort excluded; all conclusions remain the same). For the 25-month time-point the combination of the Attentional Focus and Attention Shifting scales of the Early Childhood Behavior Questionnaire (ECBQ) (Putnam et al. 2006) captured the greater range of attentional-control behaviours that toddlers are capable of.

The IBQ-R and ECBQ both use a 7-level Likert response format (ranging from ‘never’ to ‘always’). Mean scores were calculated for each scale, where a minimum of 60% of valid item responses was provided. As detailed in SM1, internal consistency for each scale of control of attention (within cohort) ranged from 0.71 to 0.87 for Duration of Orienting and Attentional Focus and from 0.56 to 0.85 for Attentional Shifting—consistent with validation samples for the original measures (Gartstein and Rothbart 2003; Putnam et al. 2006). Temperament questionnaire completion rates were greater than 80% for those infants who had reached the age of eligibility.

#### Clinical Assessment

A battery of clinical measures was used to establish diagnostic status (henceforth ‘outcome group’). At ages 2 and 3 years children were assessed using the ADOS (in BASIS Phase 1 this was using the ADOS—Generic (Lord et al. 2000), and for all other cohorts this was using the ADOS—Second Edition (Lord et al. 2012), or local translations) and parents were interviewed about their child’s adaptive development (Sparrow et al. 2005). Additionally, at the 3-year visit parents were interviewed using the Autism Diagnostic Interview—Revised (Rutter et al. 2003) (or local translations). For each cohort, clinical researchers established whether to give a diagnosis of ASD (henceforth ‘EL-ASD’) according to Diagnostic and Statistical Manual of Mental Disorders, 5th edition (DSM-5) (APA 2013) criteria—excepting BASIS Phase 1 in which ASD diagnosis was made for EL toddlers based on consensus International Classification of Diseases 10th Revision (ICD-10) (World Health Organization 1993) criteria. For the purposes of this study, EL infants not given a research diagnosis of ASD are characterised as EL-No ASD. Those without a completed assessment are characterised as EL-Outcome not known. At the point of analysis, clinical reviews were complete for 97% of the Sample 1 EL infants, and 51% of the Sample 2 EL infants; see Table 2.

**Table 2:** Parent report of autism and ADHD traits and adaptive function at age 3 years, by outcome group and phase

	SRS-2 total score (SD)				CBCL-ADHD total score (SD)				Vineland ABC score (SD)			
	TL	EL-all^a^	EL-No ASD	EL-ASD	TL	EL-all^a^	EL-No ASD	EL-ASD	TL	EL-all^a^	EL-No ASD	EL-ASD
Sample 1												
Mean SD * n*	25.99 (9.99) *94*	37.40 (23.92) *153*	30.94 (18.04) *97*	59.32 (33.92) *28*	3.18 (2.59) *84*	4.05 (2.93) *155*	3.83 (2.93) *95*	4.74 (3.32) *34*	97.75 (8.72) *61*	89.84 (10.14) *122*	91.73 (8.30)	83.62 (11.20) *29*
Sample 2												
Mean SD * n*	21.77 (10.35) *75*	40.66 (32.07) *178*	30.42 (20.09) *109*	57.44 (40.01) *68*	1.28 (1.19) *29*	2.74 (2.82) *142*	1.87 (2.18) *93*	4.46 (3.16) *48*	105.67 (7.73) *73*	94.97 (12.83) *192*	99.92 (9.25) *115*	87.30 (13.79) *76*

^a^Comprises EL-ASD, EL-No ASD and EL-Outcome not known

#### Continuous Outcome Measures at Age 3 Years

The outcome measures described below and summarised in Table 2 were available for 90–93% of Sample 1, and 42–56% of Sample 2 (largely due to not all of the sample having reached age 3 at the time of analysis). As profile discovery benefits from large sample sizes, infants were included in the latent class analysis model regardless of whether they had complete data at age 3 years (see Analytic procedure for power calculations and treatment of missing data).

##### AUTISM TRAITS

The Social Responsiveness Scale-Second Edition (SRS-2) Preschool Form (Constantino 2012) uses a 4-point scale from 0 (“not true”) to 3 (“almost always true”) across 65 items relating to autism traits. Item scores are reverse-coded where appropriate and summed to produce a total score. For missing items, we followed the SRS-2 manual recommendation of using a replacement value based on median scores for the standardization sample.

##### ADHD Traits

The ADHD DSM-oriented scale of the Child Behavior Checklist for ages 1½ to 5 (CBCL) (Achenbach and Rescorla 2001) comprises 6 statements that assess a child’s inattentive and hyperactive behaviour. Parents are asked to indicate how well each statement describes their child’s behaviour over the past 2 months on a 3-point Likert rating. Item scores are then summed to produce a total score. In accordance with the CBCL manual, missing items were treated as 0 (“Not True”) when at least 1 item rating on the ADHD scale was provided.

##### Adaptive Functioning

The Vineland Adaptive Behavior Scales, Second Edition (Vineland-2) (Sparrow et al. 2005) was used to collect parent report on their child’s behaviour in 4 domains; Communication, Daily Living Skills, Socialization, and Motor Skills, on the basis of individual items rated 0 (“Not present”) to 2 (“Fully established”). Raw scores for the sub-scales are the sum of all scores, where full credit is given for all items below the basal, and items marked as “Don’t know” or “No opportunity” are given a score of 1. Raw scores are converted to age-normed standardized scores, and the standardized scores for the 4 domains summed to give the ABC Standard Score which has a mean of 100 and a standard deviation of 15.

### Analytic Procedure

Duration of Orienting scores at 10 and 15 months, and Attentional Focus and Attention Shifting scores at 25 months were analysed without a priori allocation of ASD likelihood or outcome using LCA with repeated measures data (see SM2), in Mplus 7.4. The minimum recommended coverage proportion for LCA is 10% (Muthén and Muthén 2017). The mean coverage proportion among all the indicator variables in this study was 57%, and none fell below 46%. Descriptive plots were created in *R* 3.5.3, using the *ggplot2* (Wickham 2016) and *yarrr* packages (Phillips 2017).

LCA models were run with an increasing number of latent classes until specification of an additional class no longer improved the fit to the data, according to Sample Size Adjusted BIC (SSBIC) and the parametric bootstrap likelihood ratio test (BLRT) (see SM2). Each class number was run separately at least twice with different random starts and the output examined for local/global solutions. Models were only taken forward if it was confirmed that a global solution was reached. Where these indicators conflicted, the preferred model indicated by each fit statistic was evaluated for possible over-fitting using 2 indicators (pre-registered for Sample 2): classes of less than 3% of the entire sample would be rejected as unparsimonious; whilst if the SSBIC indicated that increasing the number of classes improved model fit beyond that indicated by the BLRT, the SSBIC scree plot would be inspected to confirm whether this was justified. If a class showed significantly lower Attentional Focus scores at age 3 years (which was not included in the class identification) than the normative class for that sample, the class was considered indicative of atypical attention development.

Distal outcomes (3-year Attentional Focus, SRS-2, CBCL-ADHD, and Vineland ABC scores) of latent class membership were estimated using the 3-step auxiliary approach for outcome measures with unequal means and unequal variances (‘DU3STEP’) (Asparouhov and Muthen 2014). Shapiro–Wilk tests indicated that raw SRS-2 scores and CBCL-ADHD were non-normally distributed (*p* < .001 in all cases). Box-Cox transformations were insufficient to achieve a normal distribution so non-transformed scores were used (a divergence from the pre-registered plan for Sample 2) using an MLR estimator, which is robust to non-normality. Power calculations computed in G*Power 3.1.9.2 (Faul et al. 2007) established that the pre-registered distal outcome analyses for Sample 2 had > 99% power to detect effect sizes indicated by the discovery sample. Post-hoc calculations showed that Sample 2 tests had > 99% power to detect the effects actually found in this sample (reported in Table 6). To ensure that Sample 2 conclusions were not distorted by missing data estimates, predictive analyses were run both with and without inclusion of infants with missing outcome data.

Exploratory Pearson’s chi-square tests of the association between likelihood or outcome group and classification to an atypical attention development class were conducted using most-likely class membership within SPSSv25. Power calculations using the Statistical Decision Tree Power Calculator (QFAB 2020) established that analyses of the effect of familial history or diagnostic status on classification to the atypical development class had > 99% power to detect a moderate effect in each sample. Post hoc calculations showed that Sample 1 analyses had 40% power to detect a significant effect of the size indicated for diagnostic status on classification to the atypical attention development class, whilst Sample 2 had 60% power.

## Results

### Model Selection

As shown in Table 3, for Sample 1 a 4-class solution was indicated, whilst for Sample 2 a 5-class solution was supported by model fit statistics (note that in both cases SSBIC appeared to support a greater number of classes but visual inspection of the SSBIC scree plot in fact confirmed the class numbers indicated by BLRT; see SM2).

**Table 3:** Model fit statistics

	1 Class	2 Class	3 Class	4 Class	5 Class	6 Class
Sample 1						
SSBIC	2342.90	2285.45	2265.35	2245.89	2243.32	
BLRT	NA	− 1161.40 *p* < .001	− 1126.39 *p* < .001	− 1110.07 *p* < .001	− 1094.05 *p* = .11	
Entropy	–	.76	.64	.72	.76	
Sample 2						
SSBIC	3311.76	3257.35	3227.72	3215.10	3200.63	3197.63
BLRT	NA	− 1644.50 *p* < .001	− 1620.16 *p* < .001	− 1588.23 *p* < .001	− 1590.10 *p* < .001	− 1560.45 *p* = .08
Entropy	–	.58	.63	.68	.76	.63

As described in SM3a, when the Swedish cohort was excluded from Sample 2 (to check that variation from the pre-registered plan in this cohort did not affect our conclusions) a 4-class solution was the best-fitting model, whereby class 4 showed the characteristics of atypical attention development equivalent to class 3 below and the main conclusions from the analyses reported below remained the same. In SM3c we report analyses conducted with Samples 1 and 2 combined. With this extended sample (*n* = 706), 6 classes were identified, which appeared to be attributable to normative attentional development being split across 2 classes. The main conclusions reported below are supported by this additional exploratory analysis. In SM3d we report results of analyses conducted with EL infants (only) from both Samples 1 and 2. With this sample (*n* = 479), 4 classes were identified, which appear to be attributable to the ‘low focus, high shifting’ class no longer being identified; all other classes were consistent with those reported below.

### Class Characteristics

#### Sample 1

**Fig. 1:**
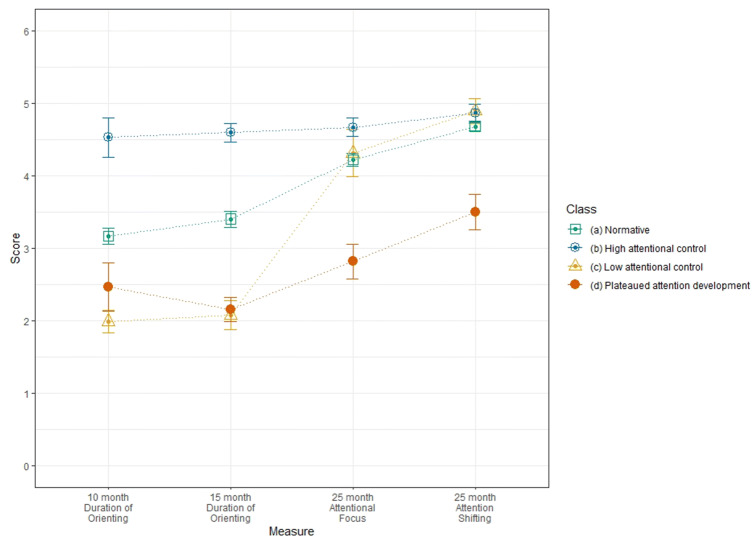
Sample means by class for the 4-class LCA model of parent report of attentional control in the first 3 years of life: Sample 1

As shown in Table 4 the majority of Sample 1 infants were assigned by the model to the same class; henceforth referred to as the normative class. Latent class was a significant predictor of 3-year Attentional Focus (χ^2^(3) = 25.72, *p* < .001), but only one class showed significantly lower Attentional Focus scores compared with the normative class; henceforth, this class is considered to show atypical attention development and, based on score profiles (see Fig. 1), is labelled the plateaued attention development class. The other classes are labelled, based on score profiles, the low attentional control class and high attentional control class; these classes are not considered to show atypical attention development. A chi-square test of association between cohort and class, using most-likely class estimates indicated that there was no significant association between cohort and class membership (χ^2^(6) = 8.51, *p* = .202, Cramer’s *V* = .12).

**Table 4:** Class counts and mean scores for parent report of attentional control

	Class									
	(a) Normative		(b) High attentional control		(c) Low attentional control		(d) Plateaued attention development		(e) Low focus, high shifting	
Sample	1	2	1	2	1	2	1	2	1	2
Class counts (and proportions) based on estimated posterior probabilities	177.44 (60.4%)	262.73 (63.8%)	47.03 (16.0%)	54.13 (13.2%)	47.03 (16.0%)	54.13 (13.2%)	20.09 (6.8%)	35.51 (8.6%)	–	18.46 (4.5%)
Class counts (and proportions) based on most-likely class membership	194 (66.0%)	297 (72.1%)	43 (14.6%)	36 (8.7%)	43 (14.6%)	41 (10.0%)	14 (5.0%)	23 (5.6%)	–	15 (3.6%)
Mean duration of orienting 10 months (SE)	3.25^b^ (0.13)	2.59^b,d^ (0.10)	4.86 (0.52)	4.48 (0.40)	1.96^a,b,d^ (0.14)	1.94^a,b,d^ (0.16)	3.42^b^ (0.35)	3.39 (0.21)	–	1.70^a,b,d^ (0.15)
Mean duration of orienting 15 months (SE)	3.48^b^ (0.18)	2.89^b,d^ (0.13)	4.71 (0.14)	4.42 (0.35)	2.05^a,b^ (0.21)	2.04^a,b,d^ (0.19)	2.48^b^ (0.39)	3.81 (0.23)	–	2.12^b,d^ (0.24)
Mean attentional focus 25 months (SE)	4.25 (0.09)	4.65 (0.08)	4.63 (0.21)	5.05 (0.19)	4.09 (0.20)	3.37^a,b^ (0.47)	2.42^a,b,c^ (0.35)	4.10^b^ (0.49)	–	2.80^a,b^ (0.29)
Mean attention shifting 25 months (SE)	4.70 (0.06)	5.10^e^ (0.08)	4.86 (0.14)	5.40^e^ (0.15)	4.49 (0.29)	3.80^a,b,e^ (0.29)	3.29^a,b,c^ (0.31)	3.62^a,b,e^ (0.28)	–	5.67 (0.18)
Mean attentional focus 3 years (SE)	4.44^b^ (0.11)	4.96 (0.08)	5.15 (0.19)	5.02 (0.63)	4.11^b^ (0.22)	4.16^a^ (0.27)	3.48^a,b^ (0.35)	3.88^a^ (0.30)	–	3.57^a^ (0.44)

Superscripts indicate which groups score higher on that measure (within the same Sample), based on significant (*p* < .05) post hoc Tukey tests for the 10-, 15- and 25-month measures and chi-square tests run within the 3-step auxiliary approach for 3-year Attentional Focus

#### Sample 2

**Fig. 2:**
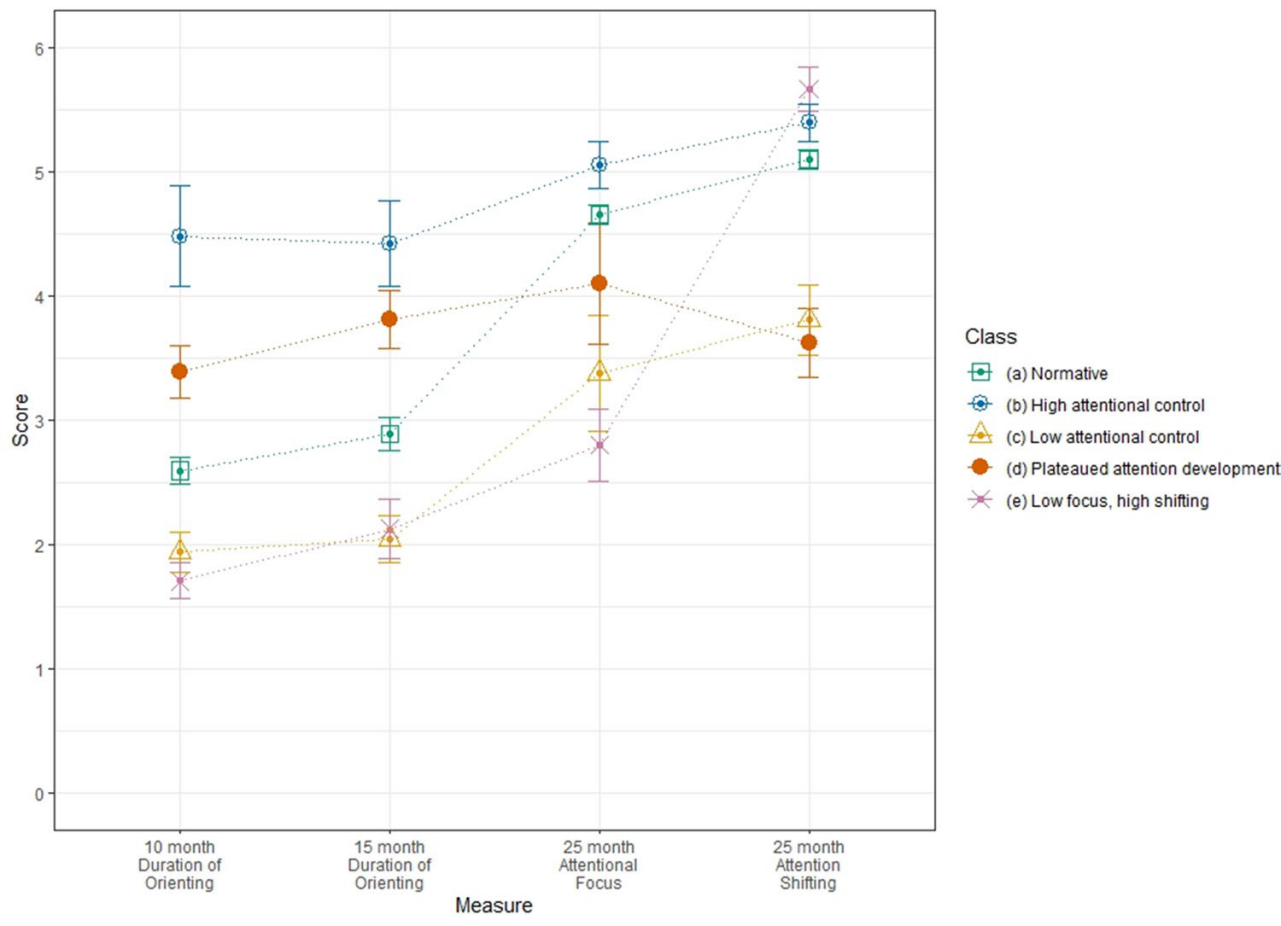
Sample means by class for the 5-class LCA model of parent report of attentional control in the first 3 years of life: Sample 2

As shown in Table 4 the majority of Sample 2 infants were assigned by the model to the same class; henceforth referred to as the normative class. Latent class was a significant predictor of 3-year Attentional Focus (χ^2^(4) = 33.61, *p* < .001) Follow-up tests for the normative class as the reference group indicated that 3 classes had significantly lower Attentional Focus scores than the normative class, and are henceforth considered to show atypical attention development: Based on score profiles (see Fig. 2), these classes are labelled the plateaued attention development class, the low attentional control class, and the low focus, high shifting class. The remaining class did not significantly differ from the normative class on Attentional Focus at age 3 years and based on score profiles is labelled the high attentional control class.

Classes did not significantly differ in terms of age at any time-point (*p* > .5 in all cases), or in terms of sex (*p* > .14 in all cases). A chi-square test of association between cohort and class, using most-likely class estimates, indicated that there was a significant association between cohort and membership of the low focus, high shifting class (χ^2^(12) = 23.74, *p* = .022, Cramer’s *V* = .14), with the odds of being classified to this class 2.0 times higher for Swedish infants than expected. Only 4 infants from across the other cohorts were classified to this group and, as shown in SM3a, when the Swedish cohort were excluded the low focus, high shifting class was no longer identified (nor was this class identified when including only EL infants from all cohorts– see SM3d); cautions over results pertaining to the low focus, high shifting class are therefore raised in the discussion below.

### Distal Outcomes of Attentional-Control Classifications

#### Autism Traits

As indicated inTable 5, latent class was a significant predictor of SRS-2 score at age 3 years, in both Samples 1 and 2.^1^ As described in SM3b, additional exploratory tests showed that main effects were not specific to the social domain (and therefore were unlikely to be driven by the conceptual overlap between items in the Attention Shifting scale pertaining to social cues). Follow-up tests comparing the atypical attention classes to the other classes indicated that the plateaued attention development class had significantly higher SRS-2 scores (indicative of elevated autism traits) than all other classes, for both Samples 1 and 2. Contrasts were significant for social and non-social domains; see SM3b.

**Table 5:** Distal outcome scores at age 3 years, and family history and diagnostic group, by latent class

	Class											
	(a) Normative		(b) High attentional control		(c) Low attentional control		(d) Plateaued attention development		(e) Low focus, high shifting		Omnibus test with full dataset [excluding infants with missing data]	
Sample	1	2	1	2	1	2	1	2	1	2	1	2
Mean SRS raw total (SE)	22.54 (1.24)	25.96 (0.93)	51.52 (9.61)	38.74 (5.17)	30.68 (4.36)	34.99 (3.19)^a^	94.58 (7.60)^a,b,c^	72.67 (8.69)a,b c,e†,	–	42.71 (12.23)	Χ^2^(3) = 151.83, *p* < .001*	χ^2^ (4) = 41.84, *p* < .001* [χ^2^ (4) = 43.76, *p* < .001*]
Mean CBCL-ADHD raw total (SE)	4.55 (0.40)	2.61 (0.26)	3.63 (0.75)	4.34 (1.37)	3.22 (0.63)	6.64 (0.89)^a,b^	8.18 (0.97)^a,b,c^	5.44 (1.21)^a,b^	–	4.96 (1.21)	χ^2^ (3) = 17.54, *p* = .001*	χ^2^ (4) = 28.07, *p* = .001* [χ^2^ (4) = 16.72, *p* = .002*]
Vineland ABC score (SE)	99.65 (1.32)	94.42 (1.04)	103.11 (3.12)	97.58 (3.33)	96.33 (3.37)	88.48 (1.91)^a‡,b^	79.35 (4.61)^a,b,c^	82.62 (4.89)^a‡,b‡^	–	89.06 (3.46)	χ^2^ (3) = 23.33, *p* < .001*	χ^2^ (4) = 13.84, *p* = .008* [χ^2^ (4) = 9.52, *p* = .049]
TL	138 (68.3%)	114 (75.5%)	18 (22.0%)	19 (12.6%)	7 (8.5%)	10 (6.6%)	1 (1.2%)	7 (4.6%)	–	1 (0.7%)^1^		
EL-all^¶^	138 (65.1%)	183 (70.1%)	25 (11.8%)	17 (6.5%)	36 (17.0%)	31 (11.9%)	13 (6.1%)	16 (6.1%)	–	14 (5.4%)^1^		
EL-no ASD	83 (66.4%)	84 (69.4%)	15 (12.0%)	8 (6.6%)	22 (17.6%)	17 (14.0%)	5 (4.0%)	4 (3.3%)^2^	–	8 (6.6%)		
EL-ASD	51 (63.0%)	22 (55.0%)	9 (11.1%)	2 (5.0%)	13 (16.0%)	6 (15.0%)	8 (9.9%)	5 (12.5%)^2^	–	5 (12.5%)		

Superscripts indicate which groups have lower SRS and CBCL-ADHD scores, or higher Vineland ABC scores, based on significant (*p* < .05) chi-square tests

*Significant after a Benjamani–Hochberg correction for 3 family-wise tests, with a false discovery rate of 5%

^†^Only when infants with missing data were excluded.

^‡^Only when the full dataset was used (i.e. not excluding infants with missing data)

^§^Proportions in each class are calculated within outcome group (i.e. for each row). Values are based on most-likely class estimates

^¶^Comprises EL-ASD, EL-No ASD and EL-Outcome not known

^1^Significant association between likelihood group and membership of the low focus, high shifting class: *p* = .025 (exploratory analysis)

^2^Significant association between ASD diagnosis and membership of the plateaued attention development class: *p* = .043 (exploratory analysis)

In terms of the additional atypical attentional control classes in Sample 2, the low attentional control class had significantly higher SRS-2 scores than the normative class. Contrasts were significant for only the non-social domain; see SM3b. The low focus, high shifting class did not show significantly different SRS-2 scores than the normative or high attentional control class.

#### ADHD Traits

As indicated in Table 5, latent class was a significant predictor of CBCL-ADHD score at age 3 years, in both Samples 1 and 2. Follow-up tests comparing the atypical attention classes to the other classes indicated that the plateaued attention development class showed significantly higher CBCL-ADHD scores (indicative of elevated ADHD traits) than the normative and high attentional control classes, for both Samples 1 and 2. As described in SM3b, additional exploratory tests showed that when items relating to attentiveness specifically were removed from the CBCL-ADHD scale, there remained a significant effect of latent class on CBCL-ADHD-modified scores, indicating that associations between development of attentional control and ADHD traits are not solely attributable to measurement overlap. In terms of the additional atypical attentional control classes in Sample 2, the low attentional control class also showed significantly higher CBCL-ADHD scores than the normative and high attentional control classes. The low focus, high shifting class did not significantly differ on CBCL-ADHD scores from the normative or high attentional control classes.

#### Adaptive Functioning

As indicated in Table 5, latent class was a significant predictor of Vineland ABC score at age 3 years, in both Samples 1 and 2. Follow-up tests comparing the atypical attention classes to the other classes indicated that the plateaued attention development class showed significantly lower Vineland ABC scores (indicative of poorer adaptive functioning) than the normative and the high attentional control classes. Although for Sample 2 the comparison between the plateaued attention development class and the normative and the high attentional control classes was no longer significant after excluding infants with missing Vineland data (due to large standard errors in the plateaued attention development class) group differences were in a consistent direction.

In terms of the additional atypical attentional control classes in Sample 2, the low attentional control class also showed significantly lower Vineland ABC scores than the normative and high attentional control classes (in the full dataset only). The low focus, high shifting class did not significantly differ on Vineland ABC scores from the normative or high attentional control classes.

### Comparison of Effect Sizes

**Fig. 3:**
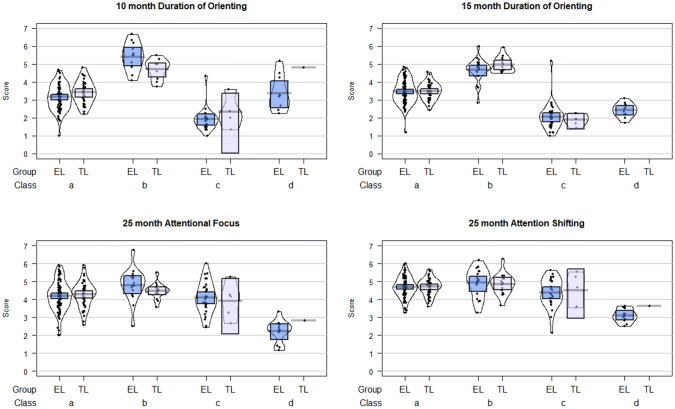
Attention scores by class and likelihood group for the 4-class LCA model of parent report of attentional control in the first 3 years of life (based on most likely class); Sample 1. *EL* elevated likelihood, *TL* typical likelihood. Class a: Normative; Class b: High attentional control; Class c: Low attentional control; Class d: Plateaued attention development

**Fig. 4:**
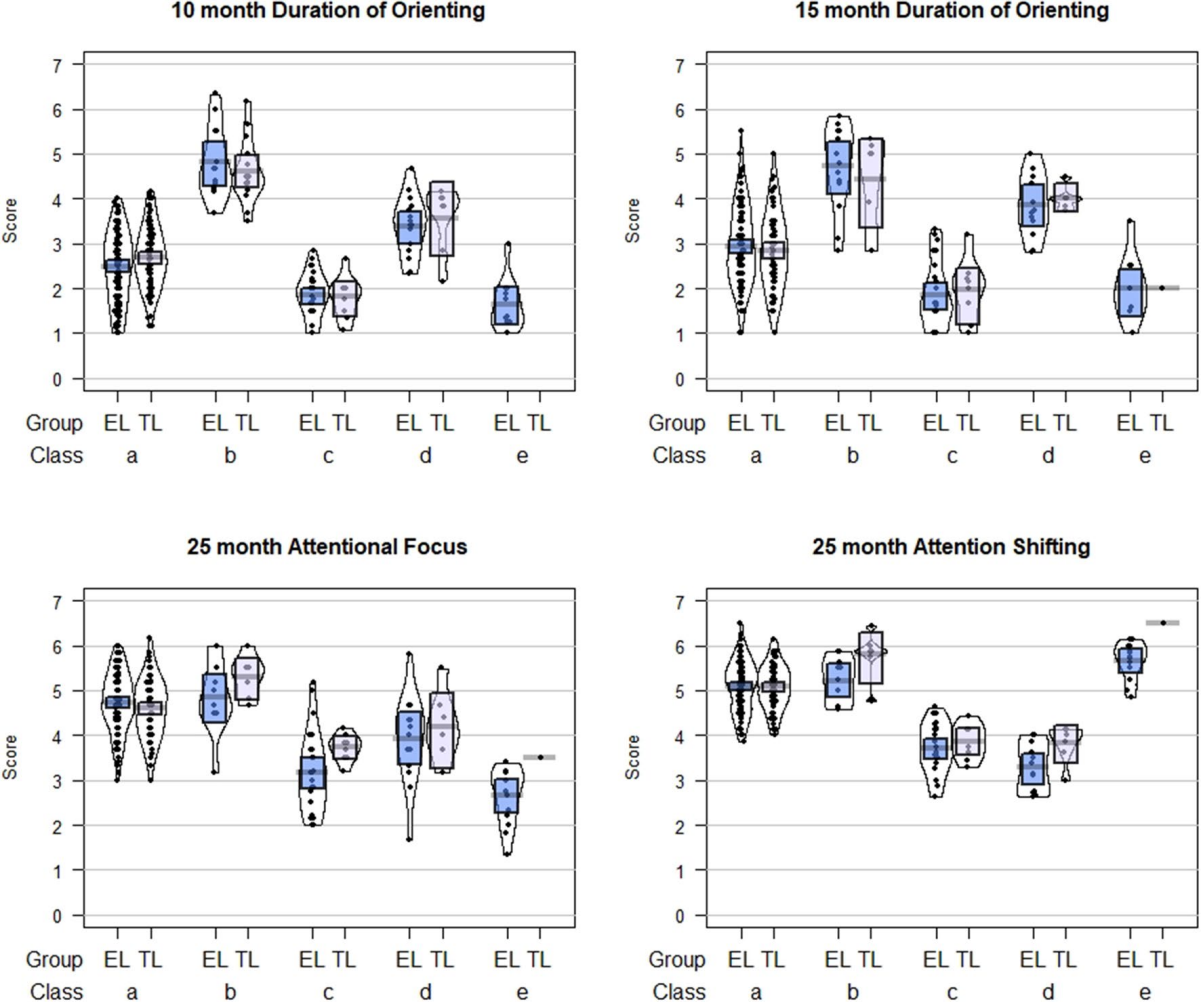
Attention scores by class and likelihood group for the 5-class LCA model of parent report of attentional control in the first 3 years of life (based on most likely class); Sample 2. *EL* elevated likelihood, *TL* typical likelihood. Class a: Normative; Class b: High attentional control; Class c: Low attentional control; Class d: Plateaued attention development; Class d: Low focus, high shifting

As shown in Figs. 3 and 4, for each measure there was some overlap between the classes, indicating that attentional profiles cannot be fully differentiated on the basis of a single measure. To establish whether the latent class approach increased the predictive value of the parent-report measures we estimated the proportion of variance explained by the latent class model, and by the parent-report measures individually, using regressions with the 3-year-outcomes as dependent variables. As shown in Table 6, the most-likely class estimate explains more variance in each of the 3-year measures than the single time-point attention scores alone, with the exception that Sample 2 Attentional Focus at age 25 months explains more variance in 3-year Attentional Focus.

**Table 6:** Effect sizes (Cohen’s d) for each of the outcome variables, with most-likely class estimate versus single-time-point attention scores as a predictor

Predictor	3-year AF		SRS-2		CBCL-ADHD		Vineland ABC	
Sample	1	2	1	2	1	2	1	2
Most-likely class estimate	.462	.569	.480	.524	.426	.515	.478	.356
Duration of orienting at age 10 months	.146	.189	.003	< .001	.026	.002	.007	.015
Duration of orienting at age 15 months	.247	.085	.003	.001	.007	.022	.016	.068
Attentional focus at age 25 months	.233	.688	.008	.013	.085	.099	.017	.015
Attention shifting at age 25 months	.076	.272	.019	.026	.123	.113	.033	.026

### Exploratory Tests of Effect of Familial History on Data-Driven Classification

A Pearson’s chi-square test based on most-likely class membership showed that there was no significant association between likelihood group and membership of the plateaued attention development class in either Sample 1 (χ^2^(1) = 3.147, *p* = .123, Cramer’s *V* = .103) or Sample 2 (χ^2^(1) = 0.405, *p* = .658, Cramer’s *V* = .031). In terms of the additional atypical attentional control classes in Sample 2, there was no significant association between likelihood group and membership of the low attentional control class (χ^2^(1) = 2.948, *p* = .091, Cramer’s *V* = .085) but there was a significant association between likelihood group and membership of the low focus, high shifting class (χ^2^(1) = 6.028, *p* = .025, Cramer’s *V* = .121), which reflects that the odds of being classified to the low focus, high shifting class was 8.5 times higher for EL infants compared with TL infants. However, since the low focus, high shifting class was largely specific to the Swedish cohort this result may not be generalizable and should be treated with caution.

### Exploratory Tests of Diagnostic Status as a Distal Outcome of Latent Class

A Pearson’s chi-square test based on most-likely class membership showed that for Sample 2 there was a significant association between ASD diagnosis and membership of the plateaued attention development class specifically for EL infants (χ^2^(1) = 4.815, *p* = .043, Cramer’s *V* = .173). This reflects that the odds of being classified to the plateaued attention development class was 4.2 times higher for Sample 2 EL-ASD infants compared with EL-No ASD infants. For Sample 1, the association between ASD diagnosis and membership of the plateaued attention development class was not significant (χ^2^(1) = 2.871, *p* = .140, Cramer’s *V* = .118), but as this test had only 40% power to detect an effect of .118 we nevertheless reviewed the odds ratios. The odds of being classified to the plateaued attention development class was 2.6 times higher for Sample 1 EL-ASD infants compared with EL-No ASD infants. In SM3 we show that in a combined sample of EL infants from Sample 1 and 2 the odds of being classified to the plateaued attention development class was 1.2 times higher for EL-ASD infants compared with EL-No ASD infants, and that the diagnostic group effect was still significant.

In terms of the additional atypical attentional control classes in Sample 2, Pearson’s chi-square tests showed that there was no significant association between ASD diagnosis and membership of the low attentional control class (χ^2^(1) = 0.022, *p* = .1.0, Cramer’s *V* = .012) or membership of the low focus, high shifting class (χ^2^(1) = 1.404, *p* = .313, Cramer’s *V* = .093).

## Discussion

We investigated attentional control as a possible intermediate phenotype of autism and ADHD, using a bottom-up analytic approach sensitive to heterogeneity in early development (as opposed to top-down grouping of infants based on familial history of autism, or diagnostic outcome). We tested whether early disruption to development of attentional control—as defined by data-driven subgroups—is predictive of more-pronounced autism and ADHD traits, and lower adaptive functioning, at age 3 years. These analyses were run first in a discovery sample of 294 infants (Sample 1), and then in a pre-registered sample of 412 infants (Sample 2).

We identified considerable heterogeneity in attention development. More classes were identified in Sample 2 than in Sample 1 (and in the combined samples than in each sample individually), as is to be expected given the difference in sample size. In both samples, the majority of infants showed the normative profile of increases in attentional control between 10 and 25 months described in the literature (Gaertner et al. 2008; Putnam et al. 2006). A minority of infants showed atypical development of attentional control: In both samples, a profile of plateaued or attenuated growth of attentional control between 10 and 25 months was associated with lower Attentional Focus scores at age 3 years compared with the normative class. In Sample 2 a class showing low attentional control across infancy, and another showing low focus and high shifting, was also identified. We describe below the atypical development classes, and their predictive associations to 3-year-outcomes, followed by a discussion of how this work contributes to our understanding of attentional control as a transdiagnostic system implicated in autism and ADHD.

### Plateaued Attention Development Class

Plateaued attention development between 10 and 25 months was characteristic of 7–9% of the samples. Classification to this profile was predictive of higher scores on clinical measures of autism (in both social and non-social domains) and ADHD traits (including when a modified scale relating to hyperactivity traits only was used) at age 3 years, relative to the normative class. The plateaued attention development profile was also associated with lower adaptive functioning at age 3 years relative to the normative class. Although comparisons no longer met significance thresholds within Sample 2 when infants with missing Vineland data were excluded, they were in a consistent direction to Sample 1 and were significant in the extended dataset described in SM3c.

Exploratory analyses indicated that infants with a familial history of autism were not more likely to show the plateaued attention development profile than infants without a familial history of autism. In Sample 2, and when EL infants from Samples 1 and 2 were combined, infants at Elevated Likelihood of autism who were later diagnosed with ASD (the EL-ASD group) were significantly more likely to be classified to the plateaued attention development profile than EL-No ASD infants; this association was not significant in Sample 1 alone, likely due to low power, but in this sample EL-ASD infants were still 2.6 times more likely to be classified to the plateaued attention development class compared with EL-No ASD infants in Sample 1. In both samples, the majority of EL-ASD infants showed normative development of parent-reported attentional control. This pattern of results echoes the trajectories of parent-reported adaptive functioning development described by Bussu et al. (2019) (based on a cohort included in Sample 1), whereby EL-ASD infants were more likely than TL or EL-No ASD infants to show a plateau in the development of adaptive functioning after the first year of life, but the majority of EL-ASD infants showed normative adaptive functioning development. In combination, these studies underscore the heterogeneity of early development in autism.

### Low Attentional Control Class

Thirteen percent of the Sample 2 showed a ‘low attentional control’ profile characterised by lower-than-average Duration of Orienting scores in infancy with some increases in absolute scores but still lower-than-average Attentional Focus and Attention Shifting at 25 months. Although a low attentional control class was also identified in Sample 1, by age 2 years this class showed similar attention scores to the normative class, and did not meet criteria for atypical development of attentional control; therefore the analyses below pertain only to Sample 2. In Sample 2, children classified to the low attentional control profile showed higher ADHD traits at age 3 years compared with the normative class, and did not significantly differ from the plateaued attention development profile in this regard. The low attentional control class also showed higher scores on clinical measures of autism traits at age 3 compared with the normative class, but had significantly lower autism traits compared with the plateaued attention development class. EL-ASD infants were not significantly more likely to show the low attentional control profile than were EL-no ASD infants. Further, exploratory analysis indicated that the association between the low attentional control class and SRS-2 scores was specific to the non-social domain (i.e. the Restricted Interests and Repetitive Behaviour subscale). Previous work has indicated that in older children, SRS scores can be inflated by non‐autism‐specific characteristics, such as internalizing and externalizing behaviour problems and developmental level (Hus et al. 2013), and additionally that there is some overlap between hyperactive-impulsive symptoms and restricted and repetitive behavioural traits in children with ADHD (Marti et al. 2014). Although follow-up research is required to confirm whether infants who show a low attentional control profile are more likely than their EL peers to be diagnosed with ADHD (and not ASD) in later childhood, our data do indicate that children in the low attentional control class do not appear to show particular difficulties with social-communication; possible interpretations of this finding are discussed below. Low attentional control was also predictive of adaptive functioning difficulties, albeit to a lesser extent than plateaued attention development.

### Low Focus, High Shifting Class

The third atypical attention development profile, which was specific to Sample 2, showed lower-than-average attentional focus at all time-points, but high attention shifting scores at 25 months. This class was characteristic of only 5% of the sample, and was primarily comprised of Swedish infants; therefore may not generalise to broader samples. Children in this low focus, high shifting profile did not significantly differ from the normative class with regards to SRS-2, CBCL-ADHD or Vineland scores at age 3 years, indicating perhaps that attention shifting may be protective against some of the difficulties indexed by these measures.

### Attentional Control as a Transdiagnostic System Implicated in Autism and ADHD

On the basis of the results presented here, and previous literature summarised by Rommelse et al. (2011) and Visser et al. (2016), we suggest that disruption to development of attentional control (which may be influenced by both genetic and/or environmental factors) is one factor which may contribute to the subsequent emergence of autism and ADHD traits. Here, disruption to attentional control may be interpreted as a vulnerability factor that interacts with variation in, for example, sensory, social or reward-processing systems to give rise to behaviours that meet thresholds for clinical concern (Johnson et al. 2015). Conversely, difficulties with attentional control may lead to the absence of a ‘protective or resilience factor’ which, if present, would enable an autistic individual to behave more in line with neurotypical expectations (Johnson 2012); although we note that performing to neurotypical expectations is not and should not necessarily be the end-goal (Bascom 2012; Fletcher-Watson and Happé 2019). In neither of these interpretations do we assume that disruption to the development of attentional control in itself causes autism or ADHD; but in both interpretations we assert that disruption to attentional control influences later behavioural presentation.

It is also possible that the direction of effects is reversed, or bi-directional, such that emergence of autism and ADHD traits disrupts the typical profile of attention development. Although the prospective study design enabled us to capture attentional behaviours before any formal diagnosis of autism was given to the El-ASD infants, nevertheless an infant on a pathway to autism or ADHD may already be experiencing atypical sensory input as early as 6 months of age (Sacrey et al. 2015), which could influence the development of attentional control. However, we note that the majority of infants with clinically-significant autism traits did in fact show a typical profile of attention development. Alternatively, a correlational association could be accounted for by some other factor. Specifically, it may be the case, and would be worth investigating in future studies, that those infants showing disruption in the attentional domain were also experiencing disruption to development across multiple other domains, either in terms of a developmental delay or regression. Indeed although as is standard in the literature we use the SRS-2 as a measure of autism traits, older children at least SRS scores may be sensitive to general behavioural and language difficulties (Hus et al. 2013). Previously, cross-domain disruption between 14 and 36 months has been observed for a sub-group of infants with later-diagnosed autism (Bussu et al. 2019; Landa et al. 2013; Sacrey et al. 2019). Should this prove to be the case it invites the question whether disruption to development of attentional control, as a domain of fundamental importance to a broad range of aspects of cognition, is itself the trigger for domain-wide disruption (Hendry et al. 2019; Johnson 2012, 2017).

### Dissociable Profiles of Attentional Control Development in Autism and ADHD

Notwithstanding our argument above that atypical attentional control is an intermediate phenotype in both autism and ADHD, our results also provide a preliminary indication that attention development follows dissociable profiles for children depending on whether they have a primary autistic or ADHD-like behavioural presentation. In our second, larger, sample, early disruption to development of attentional control—indicated by low Duration of Orienting scores from 10 months onwards—appears to be linked particularly to presentation of ADHD-relevant behaviours, in that the low attentional control profile was associated primarily with elevated CBCL-ADHD scores, and to a lesser extent (compared with the plateaued attention development profile) with elevated SRS-2 scores. Later disruption—indicated by moderate Duration of Orienting scores at 10 and 15 months, but an attenuation of the normative increase in attentional control scores at 25 months (i.e. the plateaued attention development profile)—appears to be more predictive of an autistic presentation. We propose 2 explanations for why this may be the case.

One possibility is that the timing of the disruption to development of attentional control is key to later behavioural presentation. The development of top-down control of attention in infancy has been attributed to the development of the executive attention network, which in turn may actually comprise 2 top-down control networks; a frontoparietal network which is linked closely to the orienting network in early development and therefore may be partly-functional early in development; and a cingulo-opercular network which is particularly implicated in conflict monitoring, a higher-order cognitive function that emerges at around age 2 years (Hendry et al. 2016)—for review, see Petersen and Posner (2012). It is possible that early disruption to attention development is primarily related to the frontoparietal network, and later disruption the cingulo-opercular network, and that this gives rise to dissociable behavioural presentations (Crittenden et al. 2016; Engelhardt et al. 2019). As our parent-report measures only provide a blunt index of attentional control we do not have the data to test this idea—indeed, it is challenging even with neuroimaging to dissociate these networks (Lorenz et al. 2018)—but it is an interesting question for future research.

An alternative account for why profiles of attention development seem to differ for children on a pathway towards ADHD versus autism is that both the plateaued attention development profile and the low attentional control profile are indicative of atypical development of attentional control (both profiles feature relatively-low attentional focus and shifting at age 25 months), but that Duration of Orienting scores in infancy are inflated by elevated autism traits. Specifically, an infant who shows a monotropic attentional style—characterised by a deep or intensive preoccupation for a narrow range of targets, and considered to be characteristic of many autistic children (Murray et al. 2005; Wood 2019)—is likely to score high on questions such as ‘How often did your child play with one object for 10 min or longer?’, regardless of whether they are using top-down attentional control to maintain this focus. These questions feature in both the Duration of Orienting scale of the IBQ and the Attentional Focus scale of the ECBQ (see SM1), but the latter measure also comprises questions relating to distractibility, and the ability to engage in an activity requiring attention, which are likely less-sensitive to monotropism and more-sensitive to top-down control. This would mean that infants who already have elevated autism traits at 10 and 15 months—and who might therefore be expected to score high on the SRS-2 at age 3 years—would score relatively high on the Duration of Orienting scale at 10 and 15 months, but less so on the Attentional Focus scale at 25 months—and would therefore be classified to the plateaued attention development profile. Future research should consider using measures that capture attention to a standardised range of stimuli—such as across multiple eye-tracking tasks—as a way of evaluating whether attentional profiles differ between children on a pathway towards ADHD versus autism once monotropism is ruled out as an explanation.

## Implications

Previous work from our group found that parent-report of attentional control at 14 or 24 months does not predict ASD diagnosis (Clifford et al. 2013) and that parent-report of attentional focus at 7, 14 or 24 months is not significantly associated with symptoms of ADHD in mid childhood (Shephard et al. 2019). In contrast here, using data-driven profiles informed by multiple time-points and multiple aspects of attentional control, we found that children who show plateauing development of attentional control are likely at age 3 years to show elevated autism and ADHD traits, and adaptive function difficulties—as are, to a lesser extent, children who show a profile of low attentional control. Further, we show that our LCA classes show stronger associations with 3-year outcome measures than do single attention measure scores at 2-years alone. This study may therefore benefit EL infants and their families by informing screening and intervention programmes to better equip those who most need support with coping with the day-to-day attentional demands of life. In particular this study highlights the potential value of multiple time-point screening across infancy and indicates that support is required with regard to both attentional focus and attention shifting.

With regards to improving our understanding of how variation in development of attentional control supports and restricts education, life and social-skills outcomes, we show that atypical development of attentional control in infants and toddlerhood is linked to poorer adaptive functioning by age 3 years. We suggest that disruption to the development of top-down control of attention results in a ‘double hit’ to adaptive functioning: Firstly, many day-to-day tasks require the engagement of top-down attentional control; in terms of maintaining attentional focus until the task is complete; and/or the need to switch attention between different aspects of a task. Secondly, through influencing later behavioural presentation by way of a risk or resilience factor, disruption to development of attentional control may exacerbate those aspects of the autistic phenotype that also impact on adaptive functioning (such as the ability to cope with change, and to adhere to social norms).

## Limitations, Generalisability and Future Directions

The main study limitation is that parent-reported data may be subject to inaccurate recall and a tendency for parents to interpret their child’s behaviour in line with expectations and/or their own characteristics (Rothbart and Mauro 1990). Subjectivity of report is a particular issue for infant-sibling studies such as this where, by definition, parents of EL infants will have different prior experience of behaviours relevant to the questionnaires from their older children compared with TL parents (De la Marche et al. 2015). The increased likelihood for parents of EL infants to experience attention difficulties themselves (Hughes et al. 1997; Piven and Palmer 1997) may further introduce bias. To rule out the possibility of EL-TL reporter differences driving effects, outcome-group analyses were conducted within the EL group only, and in SM3d we show that a latent class analysis of only EL infants yields similar profiles as in the full sample.

A related limitation is that some of the associations found between parent report of control of attention and parent report of autism and ADHD traits at age 3 years may be attributable to a negative halo effect, whereby children considered by their parents to show behavioural difficulties in one domain are more likely to be reported as showing challenges in other domains. The fact that, for Sample 2 (and Samples 1 and 2 combined), the plateaued attention development profile was significantly associated with diagnostic outcome, which is in part contingent on observational assessments by clinically-trained researchers, does mitigate this concern somewhat. Nevertheless, it will be important to corroborate the findings reported here by conducting data-driven classification of attentional control behaviours measured in an experimental context (for example using eye-tracking).

The findings discussed above are based on data-driven analyses in 2 large independent samples of EL and TL infants from 4 European countries, and may therefore be considered generalizable to similar infant-sibling populations. However, further research is required to establish whether different populations, such as children with a community autism diagnosis but without a family history of autism, children at elevated likelihood of autism due to having a genetic condition such as Rett’s syndrome, Neurofibromatosis type 1 or Tuberous Sclerosis Complex (Richards et al. 2015), and infants from outside the so-called WEIRD (Western, Educated, Industrialized, Rich, and Democratic) countries (Henrich et al. 2010) show similar prevalence levels of atypical control of attention (Szatmari et al. 2016).

This study reveals early differences which appear to have behavioural implications later in development. To understand if the effects of early atypicalities in attentional control development extend to the longer term, follow-up studies with children as they reach school age and beyond are required.

## Electronic supplementary material

Below is the link to the electronic supplementary material.

Supplementary file1 (DOCX 37 kb)

Supplementary file2 (DOCX 48 kb)

Supplementary file3 (DOCX 319 kb)

## Footnotes

1 Unexpectedly, the model reported more than 20% classification error for step 3 relative to step 1 for class 3. However, results were consistent when only infants with complete SRS-2 data were included (*n* = 242).
