# Supplementary material for: Atypical Development of Attentional Control Associates with Later Adaptive Functioning, Autism and ADHD Traits
Source: J Autism Dev Disord. 2020 Mar 27;50(11):4085–105. doi: 10.1007/s10803-020-04465-9 (PMC7557503; doi:10.1007/s10803-020-04465-9)
Supplement: Supplementary file 1 — Supplementary file1 (DOCX 37 kb) [file 10803_2020_4465_MOESM1_ESM.docx]

# Atypical development of attentional control associates with later adaptive functioning, autism and ADHD traits Supplementary Materials 1 – Participants and Measures: Further details

## Participants

Data for Sample 1 were collected from 3 longitudinal cohorts as part of the British Autism Study of Infant Siblings (BASIS, http://www.basisnetwork.org): ‘Phases 1-3’. Phase 1 ran from December 2006 to July 2011 and comprised 104 infants. Phase 2 ran from March 2010 to July 2015 and comprised 143 infants. Phase 3 began in September 2013 and is still ongoing. Fifty four infants, recruited for their 10-month visit prior to December 2018, were included in this discovery sample.

Of the 301 infants recruited for Sample 1, 219 (113 males) were deemed to be at EL for ASD on the basis of having 1 or more older siblings (hereafter proband) with a community clinical diagnosis of ASD. Of these EL infants, 4 had probands who were half-siblings only (in Phase 1 only). Proband diagnosis for Phases 1 and 2 was confirmed by two expert clinicians (TC – all Phases, PB – Phase 1, GP – Phase 2) based on information from the Development and Wellbeing Assessment (DAWBA; Goodman et al. 2000) and the Social Communication Questionnaire (SCQ; Rutter et al. 2003). Most probands met criteria for ASD on both the DAWBA and SCQ (Phase 1 *n =* 44, Phase 2 *n =* 77). While a small number scored below threshold on the SCQ (Phase 1 *n =* 4, Phase 2 *n =* 8) no exclusions were made due to attainment of the DAWBA threshold and expert opinion. For 21 probands (Phase 1 *n =* 2, Phase 2 *n* =19), data were only available for either the DAWBA or the SCQ, while for 9 probands (Phase 1 *n =* 4, Phase 2 *n =* 5), neither measure was available aside from parent-confirmed community clinical ASD diagnosis. Clinical review of proband scores on the SCQ and DAWBA for Phase 3 infants is ongoing.

Screening for possible ASD in the older siblings of the TL infants was undertaken using the SCQ, with no child scoring above the instrument cut-off for ASD (>15) (with missing data for 1 child at both Phase 1 and Phase 2, and screening ongoing for Phase 3).

During the period in which the parent-report measures were captured, 54 (47%) of the Phase 2 EL families took part in a randomised controlled trial (RCT) of parent-mediated intervention (Green et al., 2015; Green et al., 2017) with an additional 6 families enrolled in a similar non-RCT intervention (Green et al., 2013). Analysis was conducted to evaluate the effects of intervention (i.e. being in the treated arm of the RCT intervention or in a non-RCT intervention) on the parent report measures. As these factors showed no significant effects they were removed from further analysis.

Embedded in STAARS (Samples 1 and 2) is a randomised controlled trial (RCT) of a cognitive-training programme for infants with a familial history of ADHD, designed to target attentional control. Infants involved in either branch of the RCT (training or control) were excluded from the current study.

Data-driven classifications of attention profiles did not significantly differ in terms of age at any time-point, for either Sample 1 or Sample 2 (*p*>.5 in all cases).

## Control of attention measures

The development that takes place during the first 3 years of life, across a range of cognitive and physical skills, give rise to rapid changes in the behavioural repertoires and experiences of infants and toddlers, as is reflected in the changes in the dominant parent-report measures of attention for this age group. Thus, as noted in the main Methods section, the particular parent-report questionnaire used depended on the age of the child at that point in the study. In toddlerhood, control of attention is operationalised by the Attentional Focus and Attention Shifting scales of the Early Childhood Behavior Questionnaire (ECBQ; (Putnam et al., 2006). The Attentional Focus scale measures the child’s tendency to maintain prolonged attention to a target, and their ability to resist distraction, whilst the Attention Shifting scale indexes the ability to voluntarily shift attention, both when cued, and when endogenously motivated to do so. The sister measure to the ECBQ, designed to be more appropriate to infant behaviours, is the Infant Behavior Questionnaire-Revised (IBQ-R), which features the Duration of Orienting scale (Gartstein & Rothbart, 2003). Whilst Rothbart and colleagues conventionally recommend the use of the ECBQ for infants ages 13-17 months they note that the IBQ may be more appropriate for use with samples with (potential) developmental delays and in studies aiming to directly compare results at 13-17 months to data obtained with 3- to 12-month-olds (Putnam, 2016).

The specific questionnaires used in each cohort, at each timepoint, are detailed in Table SM1.1. As indicated in Table SM1.1, at the 15-month time-point the EASE sample completed the ECBQ (39% the full version, and 61% the Short Version) rather than the IBQ-R used by all other sites. In order to maximise comparability across samples and to best adhere with the pre-registered analytic plan (which incorrectly stated that the IBQ-R was used at 13.5-16.5 months with this cohort) 2 items were selected from the ECBQ that were identified a priori as equivalent to items in the Duration of Orienting scale; these items are highlighted in yellow in Table SM1.2.

At the 3-year time-point the BASIS Phase 3 sample completed the CBQ SF, rather than the ECBQ. Items on the CBQ AF scale were reviewed against the ECBQ AF scale (see Table SM1.2) and it was considered that these scales could be treated as equivalent. Nevertheless, for Sample 2 relevant analyses were also re-run with CBQ scores omitted to check that conclusions were unaffected: Latent class was still a significant predictor of ECBQ Attentional Focus scores (χ^2^(4)=32.75, *p*<.001) and the following classes had significantly lower ECBQ Attentional Focus scores than the normative class: the low attentional control class: (χ^2^=7.72, *p*=.005), the low focus, high shifting class (χ^2^=9.73, *p*=.002) and the plateaued attention development class (χ^2^=12.80, *p*<.001). Additionally, 28% of the EASE sample completed the full ECBQ at 36 months rather than the SF. For these participants, mean Attentional Focus scores computed from the 6 items included in the ECBQ SF were used in the final analysis. The correlation between full and SF Attentional Focus scores was *r*=.95, *p*<.001.

*Table SM1.1* Infant control of attention measures used across the contributing cohorts. Cronbach’s alpha values for each measure indicated in italics.

|  | 10 months | 15 months | 25 months | |
| --- | --- | --- | --- | --- |
| BASIS Phase 1 | IBQ Duration of Orienting (12 items) *α = .85* | IBQ Duration of Orienting (12 items) *α = .79* | ECBQ Attentional Focus (12 items) *α = .81* | ECBQ Attention Shifting (12 items) *α = .56* |
| BASIS Phase 2 | IBQ Duration of Orienting (12 items) *α = .87* | IBQ Duration of Orienting (12 items) *α = .77* | ECBQ Attentional Focus (12 items) *α = .86* | ECBQ Attention Shifting (12 items) *α = .60* |
| BASIS Phase 3 | IBQ-R SF Duration of Orienting (6 items) *α = .72* | IBQ-R SF Duration of Orienting (6 items) *α = .75* | ECBQ SF Attentional Focus (6 items) *α = .85* | ECBQ SF Attention Shifting (8 items) *α = .85* |
| EASE | IBQ-R SF Duration of Orienting (6 items)^a^ *α = .81* | ECBQ SF Attentional Focus (2 items selected for equivalence with Duration of Orienting)^b^ *α = .78* | ECBQ SF Attentional Focus (6 items)^c^ *α = .77* | ECBQ SF Attention Shifting (8 items)^c^ *α = .80* |
| Babystudie | IBQ-R Duration of Orienting (12 items) *α = .74* | IBQ-R Duration of Orienting (12 items) *α = .74* | ECBQ SF Attentional Focus (6 items) *α = .74* | ECBQ SF Attention Shifting (8 items) *α = .72* |
| ZEBRA | IBQ-R SF Duration of Orienting (6 items) *α = .77* | IBQ-R SF Duration of Orienting (6 items) *α = .71* | ECBQ SF Attentional Focus (6 items) *α = .78* | ECBQ SF Attention Shifting (8 items) *α = .69* |

ECBQ: Early Childhood Behavior Questionnaire (Putnam et al., 2006)

ECBQ SF: Early Childhood Behavior Questionnaire Short Form (Putnam, Jacobs, Gartstein, & Rothbart, 2010)
IBQ: Infant Behavior Questionnaire (Rothbart, 1981)

IBQ-R: Infant Behavior Questionnaire – Revised (Gartstein & Rothbart, 2003)
IBQ-R SF: Infant Behavior Questionnaire – Revised Short Form (Putnam, Helbig, Gartstein, Rothbart, & Leerkes, 2014)

^a^40% of the EASE sample completed the IBQ-R (12 items for the Duration of Orienting scale) at 10 months. For these participants, mean Duration of Orienting scores computed from the 6 items included in the IBQ-R SF were used in the final analysis (*α = .69)*. The correlation between 12-item and 6-item scores was *r=.*89, *p* <.001.
^b^39% of the EASE sample completed the full ECBQ (12 items for the Attentional Focus scale) at 15 months, and 61% the ECBQ SF. In order to maximise comparability across samples and to best adhere with the pre-registered analytic plan (which incorrectly stated that the IBQ-R was used at 15 months with this cohort) 2 items were selected from the ECBQ that were identified a priori as equivalent to items in the Duration of Orienting scale (see SM2 for further detail). The correlation between 12-item Attentional Focus mean scores and the mean of the 2-item subset was *r=.*75, *p* <.001. The correlation between 6-item Attentional Focus mean scores and the mean of the 2-item subset was *r=.*72, *p* <.001. In SM3a we report the results of the confirmatory tests with the Swedish data set excluded.
^c^22% of the EASE sample completed the full ECBQ (12 items each for the Attentional Focus and Attention Shifting scales) at 25 months. For these participants, mean Attentional Focus and Attention Shifting scores computed from the 6 items included in the ECBQ SF were used in the final analysis. The correlation between full and SF scores was *r=.*94, *p* <.001 for Attentional Focus and *r*=.97, *p* <.001 for Attention Shifting.

*Table SM 1.2* IBQ Duration of Orienting vs ECBQ Attentional Focus vs CBQ Attentional Focus

| IBQ-R Duration of Orienting Short Version items in bold.  Key differences in *italics.* Items equivalent to ECBQ AF highlighted in yellow. | ECBQ Attentional Focus  Short Version items in bold, reverse scored items in red. Key differences in *italics*. Items equivalent to IBQ-R DofO highlighted in yellow. | CBQ Attentional Focus  Short Version items only, reverse scored items in red. Key differences in *italics* |
| --- | --- | --- |
| How often did your baby play with one toy or object for 5-10 minutes? | **When playing alone, how often did your child play with a set of objects for 5 minutes or longer at a time?** |  |
|  | [R] When engaged in play with his/her favorite toy, how often did your child play for 5 minutes or less? |  |
| How often did your baby play with one toy or object for 10 minutes or longer? | **When engaged in play with his/her favorite toy, how often did your child play for more than 10 minutes?** |  |
|  | **[R] When playing alone, how often did your child become easily distracted?** |  |
| How often did your baby spend time just looking at playthings |  |  |
| How often did your baby look at pictures in books and/or magazines for *5 minutes* or longer at a time? | While looking at picture books *on his/her own*, how often did your child stay interested in the book for more *than 10 minutes* at a time? | **Sometimes becomes absorbed in a picture book and looks at it *for a long time****.* |
| How often did your baby pay attention *to your reading* during most of the story when  looking at picture books? | **[R] While looking at picture books *on his/her own*, how often did your child become easily distracted?** | **[R] Is easily distracted *when listening* to a story.** |
| How often did your baby look at pictures in books and/or magazines  for *2-5 minutes* at a time? | *[R]* While looking at picture books on his/her own, how often did your child stay interested in the book for *5 minutes or less?* |  |
|  | [R] When playing alone, how often did your child have trouble focusing on a task without guidance? | [R] **When *practising an activity,* has a hard time keeping her/his mind on it.** |
|  | [R] When playing alone, how often did your child move from one task or activity to another without completing any? | **[R] Will move from one task to another without completing any of them.** |
|  | **[R] When engaged in an activity requiring attention, such as building with blocks, how often did your child move quickly to another activity?** | **When *drawing or coloring* in a book, shows strong concentration** |
|  | When engaged in an activity requiring attention, such as building with blocks, how often did your child stay involved for 10 minutes or more? | When building or putting something together, becomes very involved in what s/he is doing, and works *for long periods* |
|  | [R] When engaged in an activity requiring attention, such as building with blocks, how often did your child tire of the activity relatively quickly? |  |
| How often did your baby when in a position to see the television set, look at it for 2 to 5 minutes at a time? |  |  |
| How often did your baby when in a position to see the television set, look at it for 5 minutes or longer? |  |  |
| How often did your baby stare at a mobile, crib bumper or picture for 5 minutes or longer? |  |  |
| How often did your baby watch adults performing household activities (e.g., cooking, etc.) for more than 5 minutes? |  |  |
| How often did your baby look at children playing in the park or on the playground  5 minutes or longer? |  |  |
| How often did your baby repeat the same movement with an object for 2 minutes or longer (e.g., putting a block in a cup, kicking or hitting a mobile)? |  |  |
