# Supplementary material for: Atypical Development of Attentional Control Associates with Later Adaptive Functioning, Autism and ADHD Traits
Source: J Autism Dev Disord. 2020 Mar 27;50(11):4085–105. doi: 10.1007/s10803-020-04465-9 (PMC7557503; doi:10.1007/s10803-020-04465-9)
Supplement: Supplementary file 2 — Supplementary file2 (DOCX 48 kb) [file 10803_2020_4465_MOESM2_ESM.docx]

# Atypical development of attentional control associates with later adaptive functioning, autism and ADHD traits Supplementary Materials 2 –Analytic Approach: Further details

Duration of Orienting scores at 8 and 15 months, and Attentional Focus and Attention Shifting scores at 25 months were analysed without reference to likelihood or outcome group using LCA. Technically, Likert response format ratings are ordinal data, but as the scale scores measure a continuous underlying concept, are computed from a mean of 6 or more items each, and are normally distributed, they are empirically equivalent to interval data (Carifio & Perla, 2008). Thus the latent class models were run with manifest variables treated as continuous data (i.e. LPA rather than LCA) (Muthén & Muthén, 2017). Nevertheless, as LCA is the dominant terminology in the literature, that term is used here.

An often-overlooked application of LCA is the inclusion of repeated measures data whereby the same indicators are assessed over multiple timepoints. Here, the repeated measures data available was Duration of Orienting scores (at 9 and 15 months). Unlike in latent growth models, the aim in LCA with Repeated Measures data is not to attempt to fit the data to a model (linear, quadratic or otherwise) from which one can generate slope estimates, but rather to consider how groups of people can be characterised according to time-dependent patterns (McCarthy, Ebssa, Witkiewitz, & Shiffman, 2016). Further, LCA with Repeated Measures data does not aim to identify transitions between classes over time (as in Latent Transition Analysis) but rather to investigate the characteristics of distinct developmental profiles with regards to a specific mechanism of interest (i.e. control of attention). Due to these differences, LCA with Repeated Measures is not subject to the same assumptions as latent growth curve modelling and/or Latent Transition Analysis such as normal distribution of the manifest variables and autoregressive effects of time whereby the current state is dependent on prior states.

An additional advantage of using an LCA approach over latent growth models for our research question is that repeated measures data can be combined with additional measures; in this case scores for Attentional Focus and Attention Shifting at 25 months. Extant research indicates that diagnostic group differences in control of attention may be more apparent from toddlerhood than in infancy (Clifford et al., 2013; Garon et al., 2016; Macari, Koller, Campbell, & Chawarska, 2017) meaning that critical developmental changes might be missed if only considering the infant timepoints in this study. Further, attentional shifting appears to be a particular domain of concern for ASD groups (Strang et al., 2017) but cannot be reliably captured in early infancy (Gartstein & Rothbart, 2003).

## Model fit indices

For LCA, two indicators of model fit are recommended: Sample Size Adjusted BIC (SSBIC) and the parametric bootstrap likelihood ratio test (BLRT) (Nylund, Asparouhov, & Muthen, 2008). SSBIC is of the common Information Criterion class of fit indices where, during comparison of several plausible models, the lowest value of SSBIC indicates the best fitting model. In a data simulation study comparing the performance of Information Criterion fit indices, Morgan (2015) demonstrated that AIC functioned poorly as a criterion for LCA model selection, BIC tended to underestimate the number of classes, and SSBIC performed best, particularly in conditions with smaller sample sizes and/or rare classes. Meanwhile, the BLRT uses bootstrap samples to estimate the distribution of the log likelihood difference test statistic and provides a *p*-value that can be used to compare the increase in model fit between the k-1 and k class models (models with *p*-values above an alpha of, say, 0.05 can be rejected). The BLRT has been demonstrated to control well for Type One error (i.e. is not likely to extract too many classes) across all contexts and is well-powered (>80%) to identify 4 classes with 8 continuous indicators for a sample size of 200 or more (Dziak, Lanza, & Tan, 2014). Further Dziak et al. (2014) show that with samples of 100, BLRT has 72% power to correctly select a 3 class over a 2 class model where class sizes are unequal, and under the same conditions SSBIC has 91% power.

As noted in the main Results section, for Sample 1 SSBIC appeared to support a 5 class model over the 4 class model indicated by BLRT, whilst for Sample 2 SSBIC appeared to support a 6 class model over the 5 class model indicated by BLRT, However, visual inspection of the SSBIC scree plots (see Figures SM2.1 and SM2.2) confirmed the class numbers indicated by BLRT.


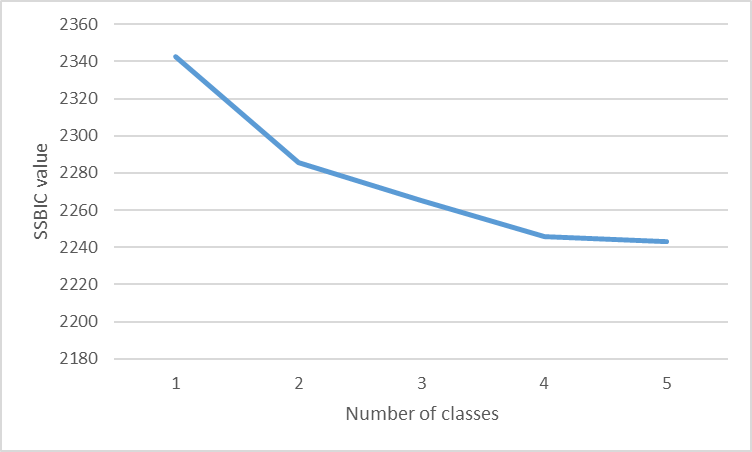


*Figure SM2.1* SSBIC Scree plot for Sample 1

*Figure SM2.2* SSBIC Scree plot for Sample 2
