# Supplementary material for: Atypical Development of Attentional Control Associates with Later Adaptive Functioning, Autism and ADHD Traits
Source: J Autism Dev Disord. 2020 Mar 27;50(11):4085–105. doi: 10.1007/s10803-020-04465-9 (PMC7557503; doi:10.1007/s10803-020-04465-9)
Supplement: Supplementary file 3 — Supplementary file3 (DOCX 319 kb) [file 10803_2020_4465_MOESM3_ESM.docx]

# Atypical development of attentional control associates with later adaptive functioning, autism and ADHD traits Supplementary Materials 3 – Additional analyses using extended and sub-samples

## SM3a: Sample 2, excluding Swedish participants

As described in the main Method section, the EASE sample completed the ECBQ (39% the full version and 61% the ECBQ SF) at the 15-month time-point rather than the IBQ as incorrectly stated in the pre-registered analytic plan. In the main results section we report the results of analysis conducted using 2 items from the EASE ECBQ data identified a priori as equivalent to items in the IBQ Duration of Orienting scale. As omitting the EASE data just from the 15-month time-point caused the covariance matrix to fall below acceptable levels, below we report the results of analysis excluding the EASE dataset entirely (*n* = 240).

### Model selection

Results of the LCA on parent report of control of attention in the first 3 years of life yielded a 4-class solution as the best fitting model – see Table SM3.1. Note that the primary information criterion for model selection appears in conflict for model 5 (i.e. acceptance of a 5-class model over 4 classes was marginally supported by SSBIC but not supported by BLRT) – however, visual inspection of the SSBIC scree plot did not support a 5-class model. Thus the 4-class solution was preferred as the more parsimonious model and is used in all subsequent analysis and discussion in this section.

*Table SM3.1* Sample 2, with EASE data excluded, Model Fit Statistics

|  | 1 Class | 2 Class | 3 Class | 4 Class | 5 Class |
| --- | --- | --- | --- | --- | --- |
| SSBIC | 1918.59 | 1863.84 | 1844.03 | 1833.90 | 1827.02 |
| BLRT | NA | -950.05  *p*<.001 | -916.90  *p*<.001 | -901.22  *p*=.01 | -890.38  *p*=.05 |
| Entropy | - | .62 | .59 | .68 | .64 |

### Class characteristics

As shown in Table SM3.2 the majority of infants were assigned by the model to the same class; henceforth referred to as the **normative class**. Two classes had significantly lower Attentional Focus scores than the normative class, and are henceforth considered to show atypical attention development: One class scored significantly lower for Attentional Focus than the normative class at age 3 years (χ^2^=7.23, *p*=.007), and on all attentional control measures from 10 months; this class is labelled the **low attentional control class**. A second class scored significantly lower for Attentional Focus than the normative class at age 3 years (χ^2^=68.17, *p*<.001) and, based on score profiles, is labelled the **plateaued attention development class.** The remaining class did not significantly differ from the normative class on Attentional Focus at age 3 years and based on score profiles is labelled the **high attentional control class.** Broadly then, these results are consistent with the main Sample results with the exception that a low focus, high shifting class is no longer identified.

Table SM3.2 Class counts and mean scores for parent report of attentional control

|  | Class | | | |  |
| --- | --- | --- | --- | --- | --- |
|  | Low attentional control (1) |  | Normative (2) | High attentional control (3) | Plateaued attention development (4) |
| Class counts (and proportions) based on estimated posterior probabilities | 45.63 (19.0%) |  | 140.015 (58.3%) | 35.09 (14.6%) | 19.27 (8.0%) |
| Class counts (and proportions) based on most-likely class membership | 40  (16.7%) |  | 158 (65.8%) | 30  (12.5%) | 12  (5.0%) |
| Mean Duration of Orienting  10 months (SE) | 2.04  (.09) |  | 2.74  (0.11) | 4.37 (0.26) | 3.69 (0.24) |
| Mean Duration of Orienting  15 months (SE) | 2.21  (.16) |  | 2.90  (0.12) | 4.30 (0.33) | 3.51 (0.20) |
| Mean Attentional Focus  25 months (SE) | 3.76  (0.19) |  | 4.67  (0.10) | 4.98 (0.19) | 3.69 (0.33) |
| Mean Attention Shifting  25 months (SE) | 3.84 (0.22) |  | 5.14  (0.07) | 5.27 (0.15) | 3.58 (0.25) |
| Mean Attentional Focus  3 years (SE) | 4.28^23^  (0.20) |  | 4.91^3^  (0.10) | 5.66  (0.18) | 3.57^1,2,3^  (0.18) |

Superscripts indicate which groups score higher for 3-year Attentional focus, based on chi-square tests run within the 3-step auxiliary approach.


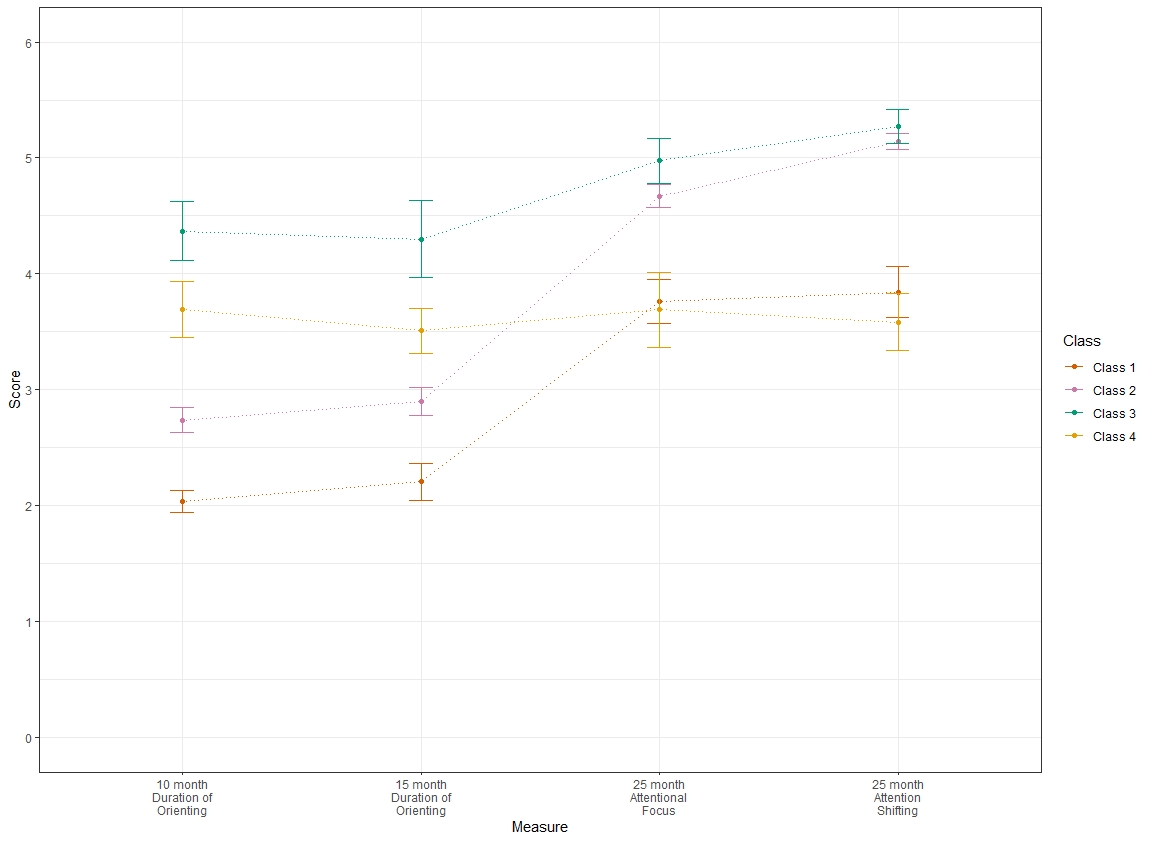


*Figure SM3.1* Sample means by class for the 4-class LCA model of parent report of attentional control in the first 3 years of life (excluding the Swedish cohort)

### Confirmatory tests of distal outcomes of attentional-control classifications

#### Autism traits

As indicated in Table SM3.3, latent class was a significant predictor of SRS-2 score at age 3 years. Follow-up tests comparing the atypical attention classes to the other classes indicated that the plateaued attention development class had significantly higher SRS-2 scores (indicative of elevated ASD traits) than all other classes. The low attentional control class had significantly higher SRS-2 scores than the normative class. These results are consistent with that of the full Sample 2.

#### ADHD traits

As indicated in Table SM3.3, latent class was a significant predictor of CBCL-ADHD score at age 3 years. Follow-up tests comparing the atypical attention classes to the other classes indicated that both the plateaued attention development class and the low attentional control class showed significantly higher CBCL-ADHD scores (indicative of elevated ADHD traits) than the normative and the high attentional control classes. These results are consistent with that of the full Sample 2.

#### Adaptive functioning

As indicated in Table SM3.3, latent class was a significant predictor of Vineland ABC score at age 3 years. Follow-up tests comparing the atypical attention classes to the other classes indicated that both the plateaued attention development class and the low attentional control class showed significantly lower Vineland ABC scores (indicative of poorer adaptive functioning) than the normative class and the high attentional control classes. These results are consistent with that of the full Sample 2. In contrast to the main Sample 2 results, neither of the atypical attention classes showed significantly lower Vineland ABC scores compared with the high attentional control class.

*Table SM3.3* Distal outcome scores at age 3 years, and family history and diagnostic group, by latent class

| 3-year outcome measure | Class | | | | Omnibus test |
| --- | --- | --- | --- | --- | --- |
|  | Low attentional control (1) | Normative (2) | High attentional control (3) | Plateaued attention development (4) |  |
| Mean SRS Raw total (SE) | 39.20 (1.92)^2^ | 27.62 (1.23) | 38.26 (5.04) | 75.38 (9.90)^1,2,3^ | χ^2^(3)=44.42, *p*<.001* |
| Mean CBCL-ADHD Raw total (SE) | 6.43 (0.74)^2,3^ | 3.90 (0.36) | 2.28 (0.72) | 6.81 (0.27)^2,3^ | χ^2^(3)=74.31, *p=*.001* |
| Vineland ABC score (SE) | 88.92 (2.14)^2^ | 97.48 (1.39) | 91.64 (5.64) | 79.11 (5.81) ^2^ | χ^2^(3)=9.29, *p*=.002* |

* Significant after a Benjamani-Hochberg correction for 3 family-wise tests, with a false discovery rate of 5%.

Superscripts indicate which groups have lower SRS and CBCL-ADHD scores, or higher Vineland ABC scores, based on significant (*p*<.05) chi-square test.

## SM3b: Exploratory checks for specificity and overlap (Sample 2)

### SRS-2

Additional exploratory tests showed that the association between attention class and SRS-2 scores reported in the main results section were not specific to the social domain There was a significant effect of latent class on both SRS-2 Social Communication and Interaction subscale scores (χ^2^(3)=39.39, *p*<.001) and Restricted Interests and Repetitive Behaviour (RRB) subscale scores (χ^2^(4)=45.33, *p*<.001). Follow-up tests showed that the low attentional control class did not show significantly higher SRS-2 Social Communication and Interaction scores than the normative class (χ^2^=2.88, *p*=.090) but did show significantly higher RRB scores than the normative class (χ^2^=19.36, *p*<.001). Meanwhile, the plateaued attention development class showed significantly higher SRS-2 Social Communication and Interaction scores than the normative class (χ^2^=9.23, *p*=.002) as well as significantly higher RRB scores than the normative class (χ^2^=6.23, *p*=.013).

### CBCL-ADHD

Additional exploratory tests showed that when items relating to attentiveness specifically were removed from the CBCL-ADHD scale, there remained a significant effect of latent class on CBCL-ADHD-modified scores (χ^2^(4)=441.11, *p*<.001). Follow up tests showed that the plateaued attention development class showed significantly higher CBCL-ADHD-modified scores than the normative class (χ^2^=147.90, *p*<.001) and high attentional control class (5) (χ^2^=6.67, *p*=.010). Additionally, the low attentional control class showed significantly higher CBCL-ADHD-modified scores than the normative class (χ^2^ = 61.79, *p*< .001) and the high attentional control class (χ^2^ = 9.04, *p* = .003).

## SM3c: Samples 1 and 2 combined: EL and TL infants

As per the pre-registration, in order to check that the main findings hold in an even larger data set, the main analyses were repeated with a combined sample of previously-unexamined data (Sample 2) and previously-explored data (Sample 1). This yielded a total sample size of 706.

### Model selection

Results of the LCA on parent report of control of attention in the first 3 years of life yielded a 6-class solution as the best fitting model, since, following the pre-registered model-selection process, the 7-class solution was rejected for containing a class of less than 3% of the entire sample; see Table SM3.4 .

*Table SM3.4:* Extended Sample Model Fit Statistics

|  | 1 Class | 2 Class | 3 Class | 4 Class | 5 Class | 6 Class | 7 Class |
| --- | --- | --- | --- | --- | --- | --- | --- |
| SSBIC | 5724.97 | 5622.48 | 5564.95 | 5620.85 | 5536.44 | 5528.88 | 5524.010 |
| BLRT | NA | -2848.95  *p* <.001 | -2789.242  *p* <.001 | -2752.017  *p* <.001 | -2734.99  *p* <.001 | -2720.84  *p* <.001 | -2708.60  *p* =.020 |
| Entropy | - | .64 | .56 | .57 | .56 | .59 | .62 |
| Minimum class size | - | 18.97% | 13.71% | 6.71% | 4.78% | 3.96% | 2.95% |

As shown in Figure SM3.2 and Table SM3.5, the largest proportion of infants (39-42%, depending on whether posterior probability or most-likely class estimates are used) were assigned by the model to the same class; henceforth referred to as the **normative class**. The second-most populated class comprised 33-35% of infants and is henceforth referred to as a **common class**. We note that in the contributing samples, the common and normative classes were combined in one single class. Latent class was a significant predictor of 3-year Attentional Focus (χ^2^(5) = 43.08, *p*<.001). Follow up tests for the normative class as the reference group indicated that the following classes had significantly lower Attentional Focus scores than the normative class: class 1: (χ^2^=5.32, *p*=0.02), class 2: (χ^2^=14.81, *p*<.001) and class 5 (χ^2^=14.25, *p*<.001); see Table SM3.4 for mean Attentional Focus within class. Henceforth, classes 1, 2 and 5 are considered atypical attention development classes and are labelled based on their score profiles as: low attentional control class (1), low focus, high shifting class (2) and plateaued attention development class (5).

### Class characteristics


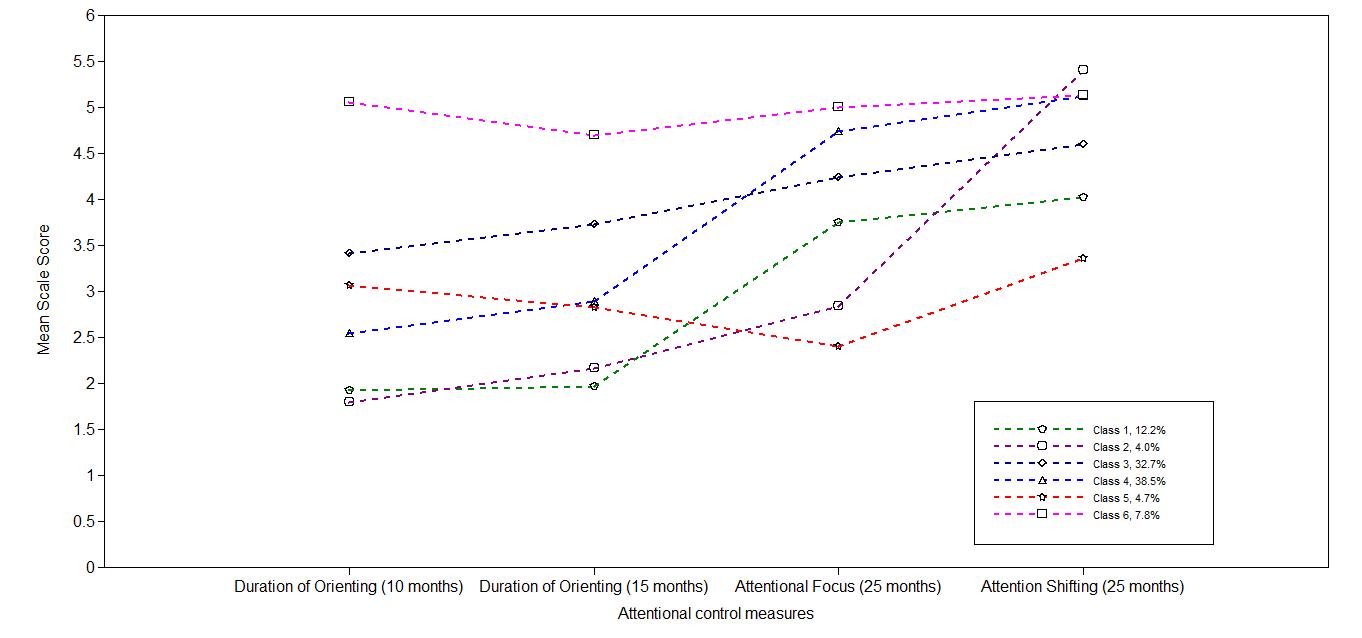


*Figure SM3.2:* Sample means by class for the 6-class LCA model of parent report of attentional control in the first 3 years of life: samples 1 and 2

Table SM3.5 Class counts and mean scores for parent report of attentional control

|  | Class | | | |  |  |
| --- | --- | --- | --- | --- | --- | --- |
|  | Low attentional control (1) | Low focus, high shifting (2) | Common (3) | Normative (4) | Plateaued attention development  (5) | High attentional control (6) |
| Class counts (and proportions) based on estimated posterior probabilities | 86.433 (12.24%) | 27.951 (3.96%) | 231.214 (32.75) | 272.143 (38.55) | 33.377 (4.73%) | 54.881 (7.77%) |
| Class counts (and proportions) based on most likely class membership | 73 (10.34%) | 19 (2.69%) | 244 (34.6%) | 300 (42.49%) | 25 (3.5%) | 45 (6.4%) |
| Mean Duration of Orienting  10 months (SE) | 1.922 (0.115) | 1.790 (0.159) | 3.418 (0.180) | 2.544 (0.194) | 3.063 (0.450) | 5.053 (0.460) |
| Mean Duration of Orienting  15 months (SE) | 1.965 (0.194) | 2.166 (0.209) | 3.727 (0.155) | 2.886 (0.174) | 2.830 (0.320) | 4.693 (0.195) |
| Mean Attentional Focus  25 months (SE) | 3.752 (0.271) | 2.835 (0.321) | 4.237 (0.191) | 4.738 (0.144) | 2.405 (0.216) | 4.997 (0.162) |
| Mean Attention Shifting  25 months (SE) | 4.017 (0.270) | 5.400 (0.290) | 4.592 (0.197) | 5.120 (0.095) | 3.364 (0.206) | 5.133 (0.169) |
| Mean Attentional Focus  3 years (SE) | 4.274^4,6^ (0.217) | 3.191^1,3,4,5,6^ (0.439) | 4.471^4^ (0.147) | 4.900 (0.110) | 3.431^3,4,5^ (0.381) | 5.165 (0.317) |

Superscripts indicate which groups score higher for Attentional Focus, based on significant (*p*<.05) chi-square tests run within the 3-step auxiliary approach.

### Confirmatory tests of distal outcomes of attentional-control classifications

#### Autism traits

There was a significant effect of latent class on SRS-2 T-Score (χ^2^(5)=313.94, *p*<.001). This remained significant after a Benjamani-Hochberg correction for 3 family-wise tests, with a false discovery rate of 5%. Follow up tests indicated that the plateaued attention development class had significantly higher SRS-2 scores (indicative of elevated ASD traits) than the normative class: (χ^2^=296.01, *p*<.001). The low attentional control class also had significantly higher SRS-2 scores than the normative class: (χ^2^=5.93, *p*=.015). In contrast, the low focus, high shifting class did not have significantly higher SRS-2 scores than the normative class: (χ^2^=0.92, *p* =.339).

#### ADHD traits

There was a significant effect of latent class on CBCL-ADHD score (χ^2^(5) = 255.68, *p*<.001). Follow up tests indicated that the plateaued attention development class had significantly higher CBCL-ADHD scores (indicative of elevated ADHD traits) than the normative class: (χ^2^=203.51, *p* <.001). The low attentional control class also had significantly higher CBCL-ADHD scores than the normative class: (χ^2^ = 5.93, *p* =.015), as did the low focus, high shifting class (χ^2^ = 4.527, *p* =.033).

#### Adaptive functioning

There was a significant effect of latent class on Vineland ABC score (χ^2^(5) = 30.88, *p*<.001). Follow up tests indicated that the plateaued attention development class had significantly lower Vineland ABC scores (indicative of poorer adaptive function) than the normative class: (χ^2^=12.39, *p*<.001). In contrast, the low attentional control (χ^2^=2.55, *p*=.110) and low focus, high shifting classes did not have significantly lower Vineland ABC scores than the normative class: (χ^2^ = 0.72, *p* = 0.40).

Table SM3.6 Mean SRS-2, CBCL-ADHD and Vineland scores at age 3 years, by latent class (all infants)

|  | Class | | | |  |  |
| --- | --- | --- | --- | --- | --- | --- |
|  | Low attentional control | Low focus, high shifting | Common | Normative | Plateaued attention development | High attentional control |
| Mean SRS Total (SE) | 45.83 (9.88) | 26.70 (3.79) | 28.74 (2.80) | 22.81 (1.24) | 102.19 (4.52) | 29.08 (4.45) |
| Mean CBCL-ADHD (SE) | 5.25 (0.65) | 4.94 (1.29) | 5.35 (0.39) | 2.17 (0.20) | 9.54 (0.48) | 2.33 (0.58) |
| Vineland ABC score (SE) | 90.84 (2.61) | 92.49 (3.83) | 97.60 (2.67) | 96.30 (1.79) | 77.79 (5.67) | 105.07 (2.92) |

Mean scores and SE calculated using 3-step auxiliary approach.

### Discussion

With this extended sample (*n* = 706) 6 classes were identified, which appeared to be attributable to the normative profile of attentional development being distributed across two groups: normative and common. As a consequence of this split the normative class in the combined sample had lower mean SRS-2 and CBCL-ADHD scores, and higher Vineland scores, than in the main sample. Consistent with the main results, 3 atypical development of attention profiles were observed, relating to low attentional control (12% of the sample), low focus but high shifting (4% of the sample), and plateaued attention development (8%). Consistent with the main results, the plateaued attention development profile was associated with higher scores on clinical measures of autism and ADHD traits, as well as lower adaptive functioning. The low attentional control profile was also associated with higher scores on clinical measures of autism and ADHD traits, but did not significantly differ from the normative class with regards to adaptive functioning. Consistent with the main results, the low-focus, high shifting class did not differ from the normative class with regards to autism traits or adaptive functioning. Results differed from the main results in that in the combined sample the low-focus, high shifting class had significantly higher CBCL-ADHD scores than the normative class but this difference seemed to be driven by the fact that the normative class for the combined sample had lower CBCL-ADHD scores than in the main sample.

## SM3d: Samples 1 and 2 combined: EL infants only

To ascertain whether classes could be driven by differences in reporter-bias between the EL and TL samples, the latent class analysis was repeated with EL infants only (from both Samples 1 and 2). This yielded a total sample size of 479.

### Model selection

Results of the LCA on parent report of control of attention in the first 3 years of life yielded a 4-class solution as the best-fitting model (acceptance of a 5-class model over 4 classes was marginally supported by SSBIC values however visual inspection of the SSBIC scree plot indicated a 4-class model, in line with the BLRT).

*Table SM3.5:* Combined Sample Model Fit Statistics

|  | 1 Class | 2 Class | 3 Class | 4 Class | 5 Class |
| --- | --- | --- | --- | --- | --- |
| SSBIC | 4950.41 | 4851.52 | 4820.13 | 3813.42 | 3810.54 |
| BLRT | NA | -2460.22  *p* <.001 | - 2401.78  *p* <.001 | - 1887.34  *p* <.001 | -1872.36  *p* =.05 |
| Entropy | - | .64 | .63 | .62 | .56 |
| Minimum class size | - | 26.62% | 10.30% | 6.29% | 9.37% |

As shown in Figure SM3.2, and Table SM3.6 the largest proportion of infants (56%-62%, depending on whether posterior probability or most-likely class estimates are used) were assigned by the model to the same class; henceforth referred to as the **normative class**. Latent class was a significant predictor of 3-year Attentional Focus (χ^2^(3)=18.13, *p*<.001). Follow up tests for the normative class as the reference group indicated that the following classes had significantly lower Attentional Focus scores than the normative class: class 1: (χ^2^=3.95, *p*=0.05) and class 2: (χ^2^=11.26, *p*=.001). Henceforth, classes 1 and 2 are considered atypical attention development classes and are labelled based on their score profiles as: low attentional control class (1) and plateaued attention development class (2). These profiles are consistent with those described in the main results section.

###
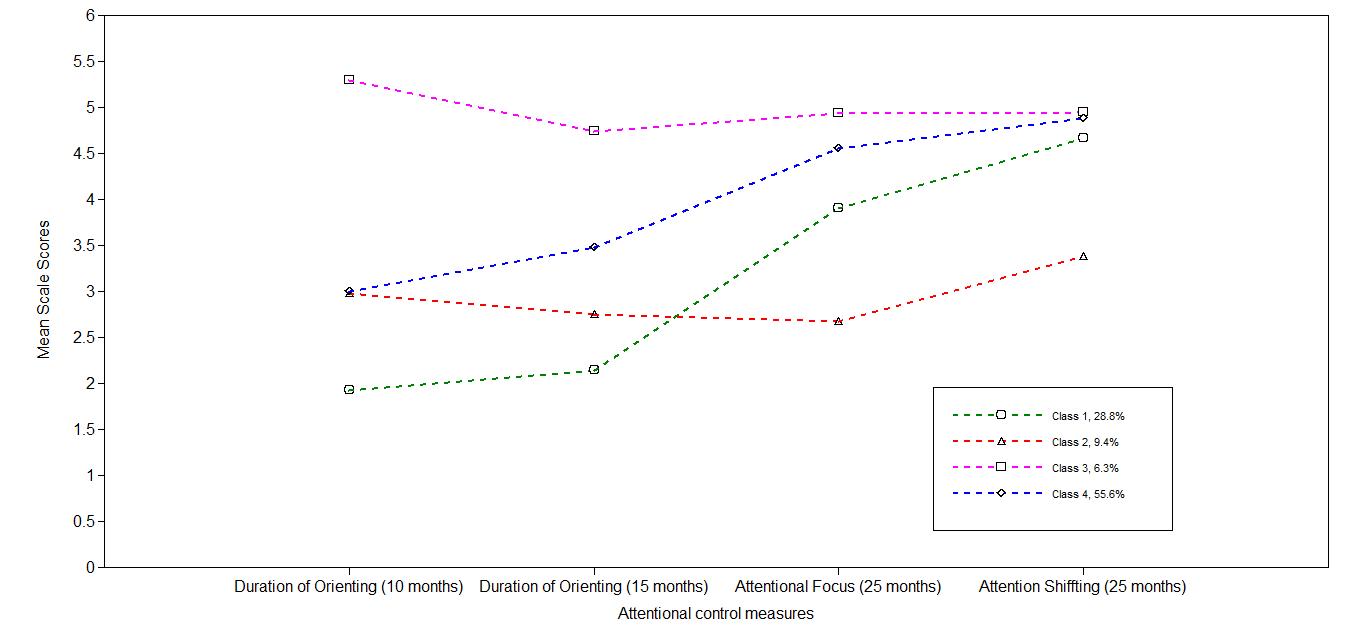
Class characteristics

*Figure SM3.2:* Sample means by class for the 4-class LCA model of parent report of attentional control in the first 3 years of life: EL infants only

Table SM3.6 Class counts and mean scores for parent report of attentional control

|  | Class | |  | |
| --- | --- | --- | --- | --- |
|  | Low attentional control class | Plateaued attention development | High attentional control | Normative attention development |
| Class counts (and proportions) based on estimated posterior probabilities | 136.34 (28.76%) | 44.39 (9.37%) | 29.80 (6.29%) | 263.47 (55.58%) |
| Class counts (and proportions) based on most likely class membership | 129 (27.22%) | 28 (5.91%) | 22 (4.64%) | 295 (6.23%) |
| Mean Duration of Orienting  10 months (SE) | 1.92 (0.12) | 2.98 (0.59) | 5.29 (0.72) | 2.30 (0.30) |
| Mean Duration of Orienting  15 months (SE) | 2.15 (0.32) | 2.76 (0.51) | 4.74 (0.18) | 3.47 (0.27) |
| Mean Attentional Focus  25 months (SE) | 3.90 (0.42) | 2.67 (0.49) | 4.94 (0.29) | 4.56 (0.08) |
| Mean Attention Shifting  25 months (SE) | 4.66 (0.34) | 3.39 (0.15) | 4.95 (0.22) | 4.88 (0.08) |
| Mean Attentional Focus  3 years (SE) | 4.18 (0.18) | 3.49 (0.33) | 4.85 (0.29) | 4.69 (0.11) |

### Diagnostic status as a distal outcome of latent class

A Pearson’s chi-square test based on most-likely class membership (see Table SM3.7) showed that there was a significant association between categorical ASD outcome and membership of the plateaued attention development class (χ^2^(1) = 4.44, *p*=.046, Cramer’s *V*=.110). This reflects the finding that the odds of being classified to the plateaued attention development class was 1.2 times higher for EL-ASD infants compared with EL-No ASD infants. This is a smaller effect than reported in the main results section, but in a consistent direction.

Consistent with the main results, a Pearson’s chi-square test showed that there was no significant association between categorical ASD outcome and membership of the low attentional control class (χ^2^(1)=1.30, *p*=.255, Cramer’s *V*=.059) for EL infants.

Table SM3.7 Number and proportion of participants in each class, by outcome group, based on most-likely class membership (EL infants only)

|  | Low attentional control class | Plateaued attention development | High attentional control | Normative attention development |
| --- | --- | --- | --- | --- |
| EL – All* | 129 (27.2%) | 28 (5.9%) | 22 (4.6%) | 295 (62.2%) |
| EL – No ASD | 71 (28.7%) | 12 (4.9%) | 10 (4.0%) | 154 (62.3%) |
| EL – ASD | 28 (23.1%) | 13 (10.7%) | 6 (5.0%) | 74 (61.2%) |

*Comprises EL-ASD, EL-No ASD and EL-Outcome not known
